# Genetic legacy and recent cross of two ancient lineages underlie the rebound of the world’s rarest primate

**DOI:** 10.1126/sciadv.adw3298

**Published:** 2026-02-11

**Authors:** Xian Hou, Shengkai Pan, Jiliang Xu, Li Hu, Weiming He, Xin Liu, Siying Huang, Zhongru Gu, Zhenzhen Lin, Yangkang Chen, Wei Li, Tao Luo, Xinrui Zhao, Qingyan Dai, Peng Cao, Feng Liu, Xiaotian Feng, Qiaomei Fu, Jiang Zhou, Jinliang Wang, Xiangjiang Zhan

**Affiliations:** ^1^ State Key Laboratory of Animal Biodiversity Conservation and Integrated Pest Management, Institute of Zoology, Chinese Academy of Sciences, Beijing 100101, China.; ^2^ Key Laboratory of Animal Ecology and Conservation Biology, Institute of Zoology, Chinese Academy of Sciences, Beijing 100101, China.; ^3^ State Key Laboratory of Efficient Production of Forest Resources, Beijing Forestry University, Beijing 100083, China.; ^4^ Cardiff University - Institute of Zoology Joint Laboratory for Biocomplexity Research, Chinese Academy of Sciences, Beijing 100101, China.; ^5^ Key Laboratory of Coastal Biology and Biological Resources Utilization, Yantai Institute of Coastal Zone Research, Chinese Academy of Sciences, Yantai, Shandong 264003, China.; ^6^ BGI-Sanya, BGI-Shenzhen, Sanya, 572025, China.; ^7^ University of the Chinese Academy of Sciences, Beijing 100049, China.; ^8^ School of Karst Science, Guizhou Normal University, Guiyang 550025, China.; ^9^ Key Laboratory of Vertebrate Evolution and Human Origins, Institute of Vertebrate Paleontology and Paleoanthropology, Chinese Academy of Sciences, Beijing 100044, China.; ^10^ Institute of Zoology, Zoological Society of London, London NW1 4RY, UK.; ^11^ Center for Excellence in Animal Evolution and Genetics, Chinese Academy of Sciences, Kunming 650223, China.

## Abstract

Despite a recent mass extinction, a few species have bounced back from the brink, but little is known about their intrinsic mechanisms. Hainan gibbons (*Nomascus hainanus*) feature a mysterious rebound, from ~13 individuals in 2003 to 42 currently. Using reliable genomic data from the fecal samples of 18 gibbons, generated through a systematically established pipeline, and from four museum specimens, our analyses reveal that Hainan gibbons have the smallest effective population size and low genetic diversity but, unexpectedly, low inbreeding and genetic load among threatened primates assessed thus far. This results from a millennial expansion mitigating bottleneck effects and a high local recombination maintaining selection efficiency. Notably, we uncover two cryptic lineages, whose recent crosses led to new family groups with increased heterozygosity. Our study reveals the importance of demographic history, genome architecture, and behavioral regulation in the recovery of endangered species and highlights the great potential of fecal genomic research in conservation biology.

## Teaser

Fecal genomic analysis of Hainan gibbon reveals roles of demography, recombination, and behavioral regulation during its recovery.

## INTRODUCTION

Although 1 million animal and plant species face extinction, mainly due to the intensified anthropogenic disturbances in recent decades (*1*), a few species have exceptionally recovered from population bottlenecks. For example, 14 critically endangered mammal species have been down listed on the Red List of International Union for Conservation of Nature from 2007 to 2025 (*2*). These successes of population recoveries are more often attributed to anthropogenic conservation efforts and inputs worldwide (*3*), but the role of intrinsic genetic and evolutionary factors in these recoveries has rarely been explored.

One major reason for this is that nearly all past studies have focused on the negative effects of historical or recent demographic bottlenecks [e.g., (*4*, *5*)] because the classical population genetic theory predicts that a species suffering from a population decline will accumulate deleterious mutations through intensive inbreeding and strong genetic drift, which, in turn, reduce fitness and may lead to an even smaller population size (*6*). Most recent genomic studies supported this assertion and found that many endangered species have suffered prolonged bottleneck, with population sizes having declined long before and remaining low and sustained through the Last Glacial Maximum (LGM) [e.g., (*4*)]. Nonetheless, species’ demographic history features not only bottlenecks but also expansions (*7*). A few studies on humans even suggested that post-LGM expansion on a millennial scale could reduce the level of inbreeding and genetic load (*8*, *9*). However, at present, we do not know the effect of post-LGM expansion on the recovery of endangered wildlife.

Gene flow is thought to be an important demographic factor that can counteract or reverse population decline (*10*). In conservation practices, human-aided gene flow (e.g., genetic rescue) has been frequently used for restoration programs of endangered species (*11*). These measures have shown their effectiveness in introducing previously unknown genetic variations through hybridization, which subsequently increases population fitness, ultimately leading to an increase in population size (*12*), although evidence also showed that some hybridization may cause outbreeding depression when immigrants are too genetically divergent (*13*). In contrast, natural hybridization such as mating between genetically different populations or lineages is commonly observed in the wild [e.g., (*14*)], but little is known about its role in the restoration of endangered animal species.

**Fig. 1.**
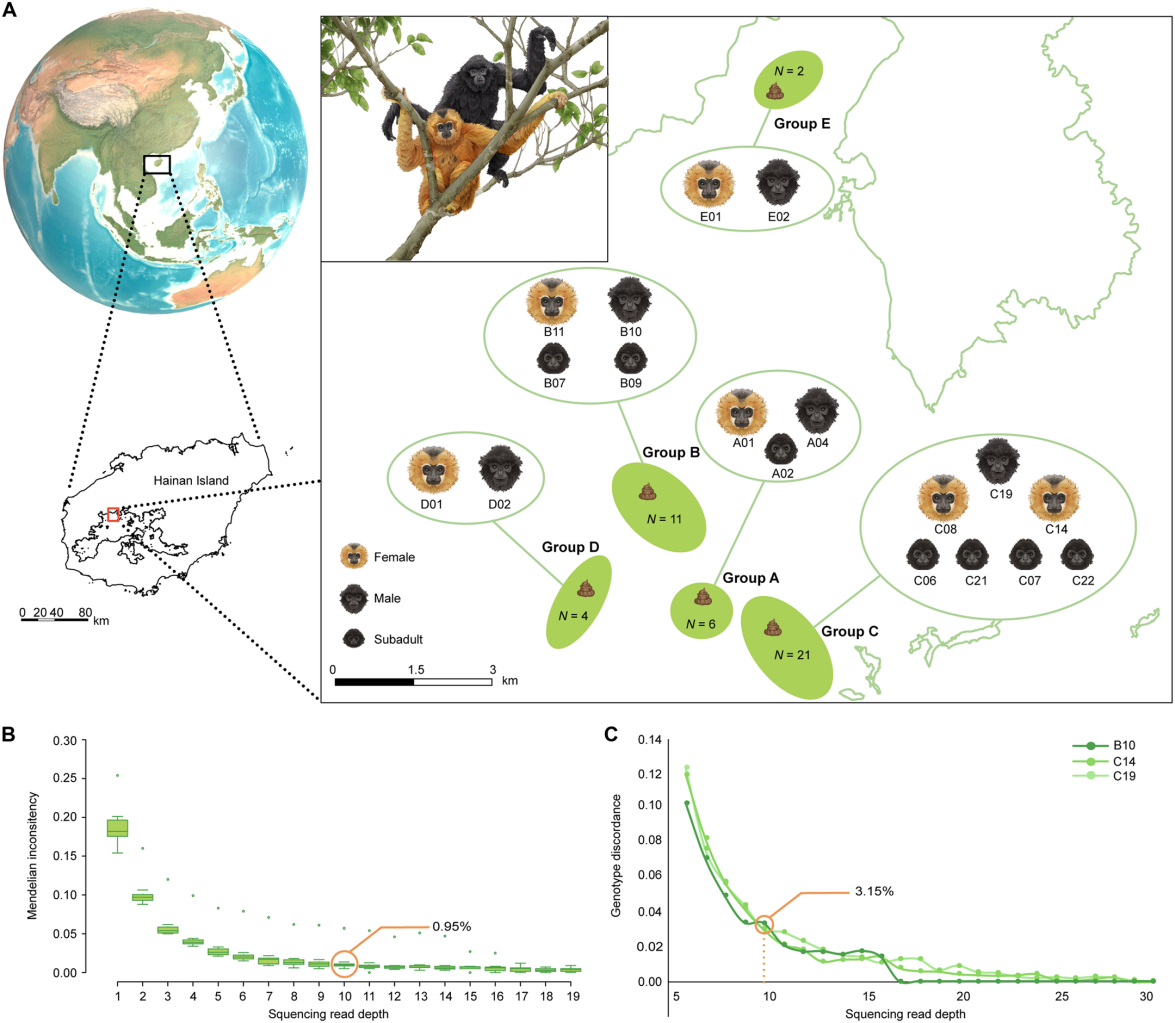
Fecal genome sequencing of Hainan gibbons. (**A**) Sampling locations, sample sizes, and identified Hainan gibbon individuals. (**B**) Allele dropout (ADO) rate estimation based on Mendelian inheritance theory (see Materials and Methods). (**C**) Genotyping discordance estimated from the sequencing data from two duplicate libraries constructed from the same gibbon individual (see Materials and Methods).

**Fig. 2.**
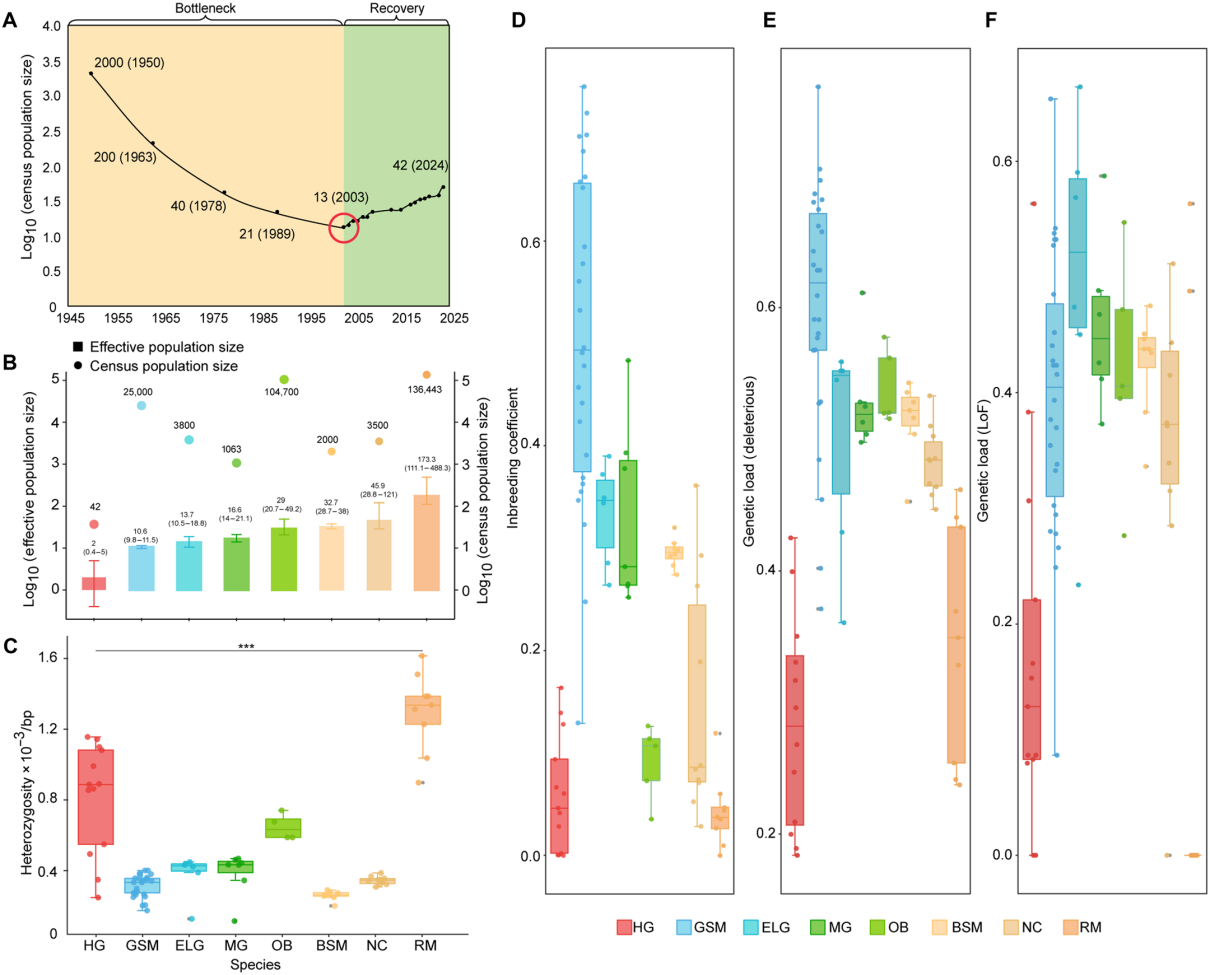
Population size and genetic status of Hainan gibbons. (**A**) Two demographic stages of Hainan gibbons inferred from previous literature (see Materials and Methods): The population decline from 1950 to 2003, followed by a subsequent population rebound. (**B**) Effective population size and census population size of Hainan gibbons (*N* = 9) in comparison with seven representatives randomly selected from the 23 primate species (data S6). The error bars display standard error. (**C** to **F**) Genome-wide heterozygosity, genome-wide inbreeding level, genetic load estimated by LoF (loss of function), and genetic load estimated by deleterious mutations in the studied Hainan gibbon population (*N* = 13) in comparison with the seven representative species. For all the 24 species, detailed information about effective population sizes, genome-wide heterozygosity, genome-wide inbreeding level, and genetic load levels estimated by LoF and deleterious mutations are provided in figs. S13 to S16 and tables S4 to S7. The box plots show the middle bar, upper bound, and lower bound representing the median, the third quartile, and the first quartile, respectively; whiskers extend to 1.5 × the interquartile range. Statistical significance was assessed using a Wilcoxon rank sum test. ***P ≤ 0.001. HG: Hainan gibbon, GSM: golden snub-nosed monkey (*N* = 26), ELG: eastern lowland gorilla (*N* = 6), MG: mountain gorilla (*N* = 7), OB: Bornean orangutan (*N* = 5), BSM: black snub-nosed monkey (*N* = 8), NC: Nigeria-Cameroon chimpanzee (*N* = 10), and RM: rhesus macaque (*N* = 9).

To address these issues, we carried out a genomics study on the recently rebounded population of Hainan gibbons (Fig. 1A). The Hainan gibbon is the rarest primate in the world, and the whole species is restricted to a ~15-km^2^ area on Hainan Island, China (*15*). The population size was roughly estimated to be 2000 individuals in the 1950s, but only 13 individuals were observed in the wild in 2003 (*15*). Fortunately, the population has had a steady growth since then (*16*), and currently, it is estimated that there are 42 gibbons in the wild. This species, thus, demonstrates a typical demographical pattern of two stages (decline and recovery; Fig. 2A) over the past decades (see Materials and Methods) and provides an unprecedented opportunity to explore the roles that genetics has played in the recovery of a critically endangered species.

However, obtaining high-quality DNA samples (e.g., blood and tissue) from endangered primates such as Hainan gibbons in the wild is nearly prohibitive due to inaccessible habitats (e.g., canopy in the primary forest), few sightings, disturbance to their natural behavior, and ethical issues (*17*). Therefore, in this study, we collect the Hainan gibbon feces from the wild and establish a systematic next-generation sequencing (NGS) pipeline for fecal genome-wide data generation, quality assessment, and genotyping error estimation [e.g., allele dropout (ADO) rate]. By combining current fecal genomic data and historical museum genomic data, we obtain population genomic data spanning two distinct demographic stages (decline and recovery) of the Hainan gibbon population. Here, we use this research system to address two questions: (i) Which role the long-term demographic history has played during the recent population rebound of the Hainan gibbon, and (ii) whether and how natural gene flow has contributed to this rebound? Our study reveals a positive role of a post-LGM expansion in mediating the negative genetic consequences of a prolonged LGM-associated bottleneck. In addition, we find the contributions of high local recombination in the Hainan gibbons’ genome in alleviating the genetic load and the important role of the interaction between balancing selection and local recombination in maintaining functional variation in this small population. Furthermore, we highlight a prominent role of the recent natural cross of two ancient lineages during the rebound of Hainan gibbons.

## RESULTS

### The generation of fecal genomic data of Hainan gibbons

To obtain reliable NGS genomics data from fecal samples, we developed a systematic pipeline for sample collection, host DNA enrichment, sequencing, data quality assessment, and genotyping error estimation (fig. S1). Because Hainan gibbons are an elusive species living in dense tropical rainforest, we collected 42 fresh fecal samples covering the current distribution of Hainan gibbons to obtain genetic information from as many individuals as possible (Fig. 1A). In total, we identified 18 gibbon individuals in the five extant family groups using the microsatellite system we developed (*18*). These samples constitute ~40% of the entire gibbon population, one of the highest percentages of individuals assessed for existing animal population genomics studies.

For each individual, we enriched the gibbon DNA from the fecal samples using a cytosine-phosphate-guanine (CpG) methylation–based enrichment method (figs. S1 and S2; see Materials and Methods) (*19*) with an average enrichment fold of 20 (ranging from 0.5 to 152; data S3). The post-enriched DNA samples were sequenced with an average of 1163 million (M) paired-end short sequencing reads generated for each individual (data S4). Mapping these reads to the *Nomascus leucogenys* reference genome retrieved an average of 133 M (23 M to 572 M) clean reads per individual, giving an averaged genome coverage of 46.67% (17.32 to 84.43%; data S4) and an average sequencing depth of 3× (1.2 to 10.7×) after removing duplicates. Using the Genome Analysis Toolkit (GATK) pipeline, we identified a total of 2,155,565 single-nucleotide polymorphisms (SNPs) from the sequenced gibbon population with each individual containing 875,824 SNPs on average (328,131 to 1,260,483; data S4). We further extracted DNA from four museum samples (see Materials and Methods) and generated an average of 529 M reads for each individual, from which we identified 293,892 SNPs on average (10,339 to 631,058; data S5).

NGS-based genotypic data generated from noninvasive genetic samples such as feces may have an inherent high ADO rate due to a low proportion of host DNA and low sequencing depth (*19*); however, up to date, there are no systematic studies on the control and assessment of these genotyping errors. In this study, we systematically assessed the ADO rate through Mendelian inconsistency and genotype discordance analyses (fig. S1; see Materials and Methods). We checked the relationship between ADO and SNP depth and observed that the ADO trend levels off at 10× sequencing calling depth (Fig. 1, B and C), with a very low ADO rate (Mendelian inconsistency: 0.95%; genotype discordance: 3.15%). We also checked the relationship between the number of usable SNPs and varied sequencing calling depth (6×, 10×, 15×, 20×), taking account of its effects on related genetic estimation. Again, the 10× SNP dataset demonstrated a sufficient number of usable SNPs and a minimized ADO rate (figs. S4 to S8, and the Supplementary Materials), which therefore was used for downstream analyses. In addition, we assessed whether the methylation-based enrichment method introduced bias in guanine-cytosine (GC) content in our fecal genomic data. No significant difference in mean GC content was observed between the fecal genomic data and those derived from museum samples (fig. S9), suggesting that the enrichment method did not induce GC content bias. Furthermore, sequencing reads enriched from fecal samples were normally distributed across genomic windows (fig. S10), suggesting no substantial bias in genomic representation of the sequenced fragments. Together, these results demonstrate that the fecal genomics data of the Hainan gibbon are reliable for subsequent genetic analyses.

### The current genetic status of Hainan gibbons

The identified fecal genomic SNPs enabled us to construct a relatively complete pedigree for 16 Hainan gibbons covering all five family groups and spanning three generations (fig. S11). Thirteen individuals are assigned to the population decline stage (1950 to 2003) and three to the recovery stage (after 2003) (Fig. 2A and fig. S12; see Materials and Methods). During the decline stage, the effective population size (*N*_e_) based on the linkage disequilibrium (LD) method was estimated to be 2 (0.4 to 5.0), the smallest *N*_e_ among primate species with population genomic information available (Fig. 2B and data S7). Heterozygosity estimation showed that Hainan gibbons, comparable to other threatened primates, have a low level of genetic diversity at the genome level (Fig. 2C, fig. S13, table S4, and data S7). These population demographic and genomics estimates, thus, agree well with previous assertions about Hainan gibbons, the world’s rarest primate (*18*).

Population genetics theory predicts that smaller *N*_e_ will result in higher levels of inbreeding and more elevated genetic load in populations of endangered species (*6*). We were thus interested in knowing whether this is the case for Hainan gibbons. Unexpectedly, during the population decline stage, our FEstim analysis (*20*), which is based on the probability of identity by descent for two alleles in an individual, revealed that the Hainan gibbon exhibits one of the lowest inbreeding levels among the 19 studied threatened primate species (Fig. 2D, fig. S14, table S5, and data S7), and this inbreeding level was comparable to most unthreatened primates. Furthermore, by checking the loss-of-functional (LoF) mutations and deleterious mutations in the homozygous state (see Materials and Methods), we found that Hainan gibbons exhibit one of the lowest levels of genetic load among the threatened primates and are similar to those of most unthreatened primates (Fig. 2, E and F; figs. S15 and S16; tables S6 and S7; and data S7). Hainan gibbons, therefore, present a puzzle that a critically endangered species, featuring lowest census and effective population sizes and low genomic diversity, has low levels of inbreeding and genetic load among the endangered primate species assessed so far.

### A millennial population expansion mitigated the deteriorated genetic effect of the prolonged LGM-associated bottleneck

**Fig. 3.**
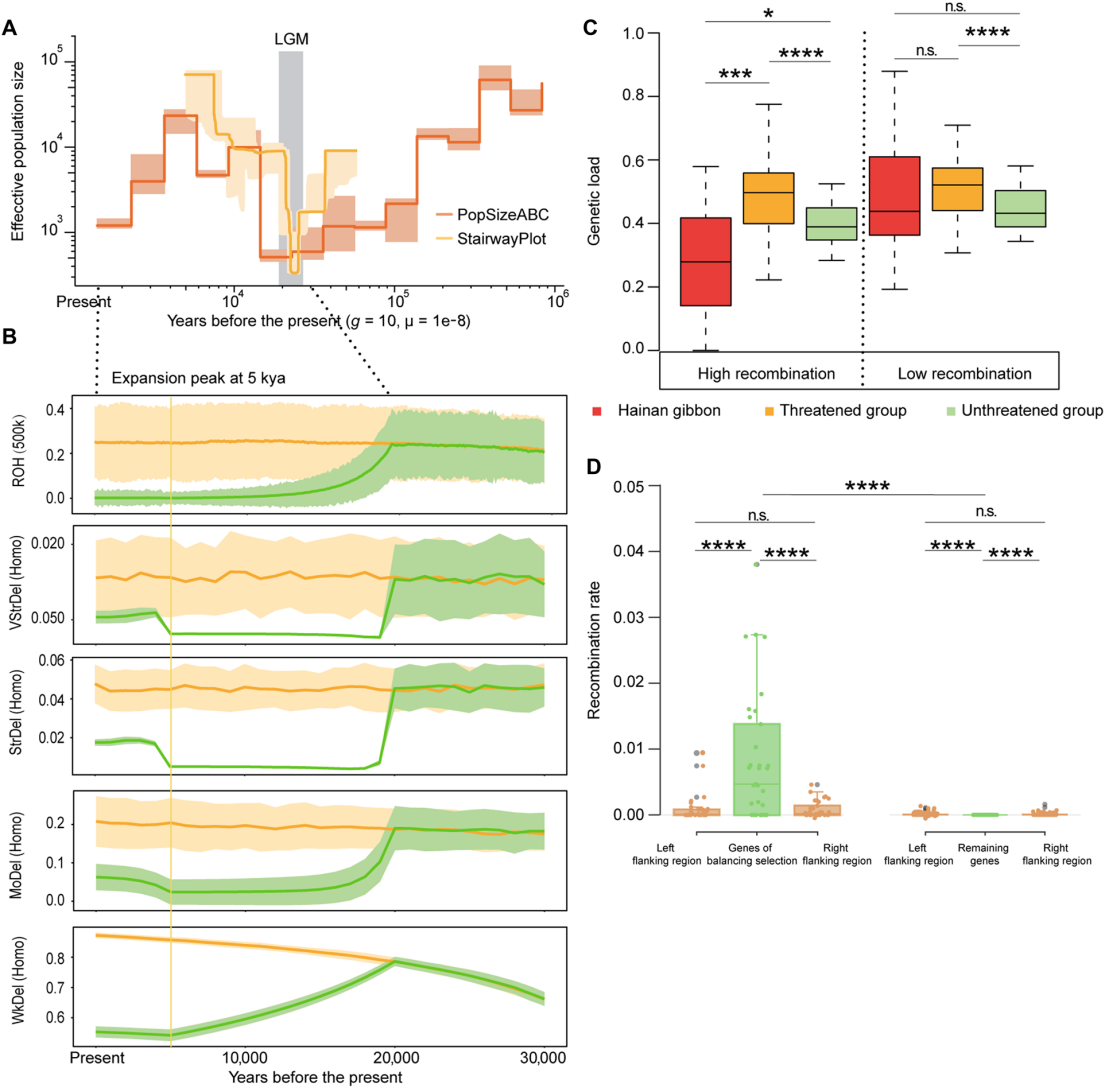
Genetic effects of the millennial population expansion and high local recombination of Hainan gibbons. (**A**) Demographic history reconstruction of Hainan gibbons using PopSizeABC and Stairway plot. (**B**) Simulated accumulation trajectories of inbreeding and genetic load levels based on the gibbon demographic history inferred in this study (green) or the island small population model without the recent 5-kya expansion (orange). The solid line and shade denote the median and 95% confidence interval (CI) of the inference in (A) and (B). ROH (runs of homozygosity) symbolizes the inbreeding level, and Homo denotes deleterious mutations in homozygous state. VStrDel, StrDel, MoDel and WkDel mean very strong (*s* < −0.1), strongly (−0.1 < *s* < −0.01), moderately (−0.01 < *s* < −0.001), and weakly deleterious mutations (*s* ≥ −0.001), respectively. (**C**) Genetic load comparisons between frequently (high GC4) and rarely recombining (low GC4) genome regions in Hainan gibbons compared with threatened and unthreatened primates. Statistical significance was assessed using a Wilcoxon rank sum test. (**D**) Recombination rate comparisons between genes with or without balancing selection and their flanking regions (10 kb). Statistical significance was assessed using a paired *t* test (between left and right flanking regions) and a Wilcoxon rank sum test (between balancing- and nonbalancing-selected genes). The box plots show the middle bar, top bound, and bottom bound representing the median, the third quartile, and the first quartile, respectively; whiskers extend to 1.5 × the interquartile range. *P ≤ 0.05, ***P ≤ 0.001, ****P ≤ 0.0001, and n.s.: no significant difference.

**Fig. 4.**
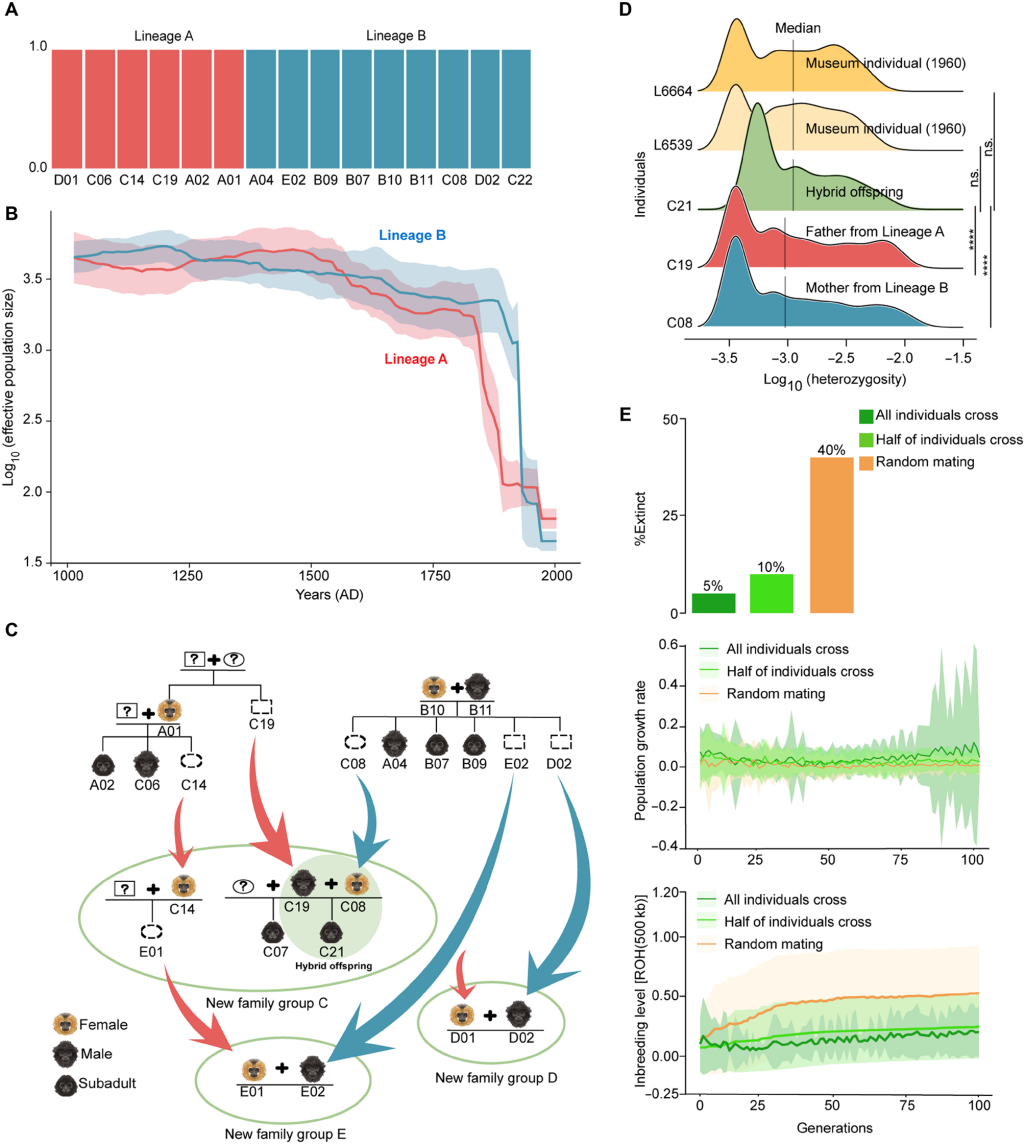
Identification of two cryptic genetic lineages from the current Hainan gibbon population and evolutionary outcomes of their cross. (**A**) Population structure of lineages A and B of Hainan gibbons. The individuals in the decline stages were used in this analysis (see Materials and Methods). (**B**) Recent demographic history of the Hainan gibbon population within 100 generations was reconstructed by GONE. The solid line and shaded area denote the mean and 95% CI of the inference, respectively. (**C**) The pedigree of the studied Hainan gibbon population constructed using nuclear genomic SNPs. Rectangular boxes and circles represent males and females, respectively. Question marks mean unknown individuals: unsampled or uncertain existence. Boxes with dashed lines represent emigrants. Red and blue arrows denote immigration events from lineages A and B, respectively. (**D**) Comparisons of genome-wide heterozygosity between the hybrid offspring, its parents, and museum individuals. Statistical significance was determined using a Wilcoxon rank sum test. ****P ≤ 0.0001 and n.s.: no significant difference. (**E**) Effects of lineage cross events on the extinction rate, population growth, and inbreeding level of the Hainan gibbon population over the next 100 generations. All individuals cross and half individuals cross means that all or half of gibbon individuals mate with individuals from different lineages, respectively. Random mating means that one individual mates with another individual randomly. The solid line and shaded area denote the mean and 95% confidence interval of the inference, respectively.

To understand whether historical demography has contributed to the low levels of inbreeding and genetic load found in the Hainan gibbons, we first reconstructed the gibbon demographic history from 1 million years ago (Ma) to 50 years ago using multiple methods (Figs. 3A and 4B and fig. S17). Our analysis inferred that the effective population size of Hainan gibbons decreased from ~1 Ma and reached a minimum around the LGM. Since then, the population increased and peaked 5 thousand years ago (kya), followed by a decline until the present. As a result, unlike most endangered species reported previously, which typically experience a long-term continuous decline [e.g., (*4*)], Hainan gibbons had a unique recent expansion at 5 kya.

Next, we used a forward-in-time simulation to reconstruct the accumulation trajectories of inbreeding [runs of homozygosity (ROH)] and genetic load (the ratio of different deleterious mutation types in homozygous state) for Hainan gibbons based on two different demographic scenarios: the one inferred for the Hainan gibbon in the present study and a classical island population history without the recent 5-kya expansion (a continuous decline from LGM to the present; see Materials and Methods). Our results showed that, along with the increase in *N*_e_ after the LGM (Fig. 3A), the inbreeding and genetic load of the Hainan gibbon had been reduced (Fig. 3B and fig. S18), and the lowest values occurred consistently at 5 kya (e.g., 0.08% of its LGM ROH estimate; Fig. 3B and fig. S18). Since then, although genetic load had slightly increased when the *N*_e_ had decreased again, the present level of genetic load is much lower than expected under the scenario of a classical island population history (Fig. 3B). This evidence suggests that a recent substantial *N*_e_ increase at 5 kya helped the Hainan gibbons in mitigating the negative genetic consequence of a prolonged LGM-associated bottleneck and resulted in sufficient genetic variations to cope with the most recent population decline from 1950 to 2003.

### High local recombination helped to reduce the genetic load and maintain functional variation

It has been suggested that recombination can help remove harmful mutations by improving the efficiency of purging selection (*21*), but there is no study on the role of recombination during the endangerment and recovery of threatened species. To address this issue, we characterized the local recombination patterns in the Hainan gibbon genomes. Using GC content at fourfold degenerated sites (GC4) as a proxy of local recombination rate (*22*), we calculated the GC4 content for each of the annotated genes (*N* = 22,003 on average) in the 24 primate species (see Materials and Methods). To investigate the effect of local recombination in alleviating the local genetic load in the Hainan gibbons, we divided the gene set into the high and low recombination (GC4) groups. Within each group, we compared the genetic load of Hainan gibbons against those of threatened (*N* = 18) and unthreatened primates (*N* = 5) (see Materials and Methods). We found that the genetic load of the Hainan gibbon was significantly lower than that of other threatened primates, especially in high recombination regions, and even found that these estimates for the Hainan gibbon were even significantly lower than those of the unthreatened primates (Fig. 3C). This pattern is consistent with the observation that the Hainan gibbon exhibits significantly higher GC4 levels in high-recombination regions compared with other primates (*P* < 0.05 for all comparisons; Wilcoxon rank sum test).

To understand whether high local recombination facilitates the maintenance of functional variation in the Hainan gibbon population, we calculated ß (*23*), a metric that identifies clusters of alleles at similar frequencies to detect the site under balancing selection across the genome (see Materials and Methods). We identified a total of 503 loci subjected to balancing selection, of which 28% were located in genic regions (22 genes). Next, we examined the recombination rate in these balancing-selected genes (see Materials and Methods) and compared them to with their flanking genomic sequences (10 kb) and to the remaining (nonbalancing-selected) genes. The recombination rates in the balancing-selected genes were significantly higher than those in the flanking regions or the remaining genes (Fig. 3D), suggesting that high recombination likely improves the overall selection effectiveness. Our results indicate that recombination may play an important role in maintaining high variations in genes of adaptive relevance in a small population.

Among the 22 selected genes identified in the Hainan gibbon population, we identified two genes, *TEKT3* and *CCT6B*, which are related to reproduction (data S8). *TEKT3* encodes proteins responsible for sperm flagellar formation, which are critical for sperm mobility and subsequent fertilization (*24*). The loss of *CCT6B* leads to a decreased ratio of normal sperm (*25*). In addition, we detected balancing selection signatures in genes associated with carbohydrate metabolisms (*βGLU*) (*26*). Given that the nutrition compositions of Hainan gibbons vary in different seasons (carbohydrates and crude fiber dominant in dry seasons while proteins and lipids dominant in wet seasons) (*27*), balancing selection on this metabolism-related gene might enable Hainan gibbons to adapt to dietary shifts among seasons. Together, our results show that most of the genes under balancing selection in the Hainan gibbon are related to either the survival or reproduction (data S8), which may have contributed to the survival of the last Hainan gibbon population during the most recent population decline.

### Natural cross of two old lineages promotes the rebound of Hainan gibbon population

Ecological surveys and behavioral studies have a consensus that only one population of Hainan gibbons remains in the mountain area of central Hainan Island (*15*). Unexpectedly, our analyses of nuclear genomic SNPs detected two cryptic but distinct lineages from the extant population. Within the two lineages, the family groups A and B are known to be the oldest in the extant population because only these two groups survived in 2003 (*15*). Population structure, principal components analysis (PCA), and phylogenetic analyses based on fecal genomic SNPs further showed that all individuals in the two groups could be assigned accurately into each of the two genetic lineages (Fig. 4A and figs. S20 and S21), which were largely consistent with the two mitochondrial haplotypes dominant in group A or B, separately, except for one individual (B11) (fig. S22 and data S9). Therefore, we named the two lineages as lineages A and B hereafter. Demographic history reconstructed from LD in SNP data showed the differentiation in *N*_e_ trajectories between the two lineages (Fig. 4B). To estimate the timing of this divergence, we applied a coalescent hidden Markov model (CoalHMM) simulation (*28*) and found that the two lineages separated around 450 years ago (~157 to 576 years ago) (fig. S23).

Previous studies posited that one of the most notable signs of the population rebound of Hainan gibbons is the formation of family group C in 2011 (*16*). This was thought to be a recolonization event because a previous family, inhabiting the same habitat, had possibly been poached before 2003 (*15*). Current founders of group C were therefore speculated to be immigrants from the two oldest groups (A and B) (*18*). However, until now, there is no direct evidence either to prove or to dispute this speculation and the genetic consequence of this group formation. In the present study, with the reconstructed pedigree, we found that the founders of group C indeed came from the two different genetic lineages (Fig. 4C). Our fastsimcoal simulation further verified that there was no gene flow between lineage A and B from their separation until the formation of group C (figs. S24 and S25 and table S8; see Materials and Methods). Therefore, we conclude that group C was founded by emigrants from groups A and B, which started their divergence about 450 years ago.

We then explore the genetic consequences of the lineage cross due to the formation of group C. We first compared the expected heterozygosity (*H*_e_) of group C with groups A and B. Our result showed that the expected heterozygosity of group C [*H*_e_ = 0.37, 95% confidence interval (CI): 0.3697 to 0.3703] is higher than that of group B (*H*_e_ = 0.33, 95% CI: 0.3293 to 0.3307) but closer to group A (*H*_e_ = 0.38, 95% CI: 0.3767 to 0.3833). Although group C was formed only about 10 years ago, based on the reconstructed Hainan gibbon pedigree (Fig. 4C and fig. S11), our structure analysis already identified one hybrid offspring (C21), whose parents (C08 and C19) were inferred to come from lineage B and Lineage A, respectively (fig. S26). Therefore, we compared the individuals’ genomic heterozygosity with its parents. As expected, the heterozygosity of C21 is higher than that of its parents (*P* < 0.0001; Wilcoxon rank sum test) and is similar to the heterozygosity of two museum gibbon individuals collected in 1960 (Fig. 4D), which had lived before the most recent population decline started in 1950s (*15*). We further compared the heterozygosity of purebred offspring (B07, B09, C08, A04, E02, and D02) whose parents were from the same lineage (Fig. 4A) with that of hybrid offspring C21. The results showed that the hybrid offspring had higher heterozygosity than the purebred offspring (fig. S27). Given that a higher genetic diversity implies heterosis (*10*) in the short term and more evolutionary potential in the long run, our results suggest that the natural cross between gibbons from the two cryptic lineages in group C may offer genetic benefits to the Hainan gibbon population, potentially contributing to its gradual recovery since 2011.

In addition to group C, we also observed lineage cross events in the most recent family groups D and E (Fig. 4C) formed in 2015 and 2019 (*16*), respectively. These events involved breeding pairs from different lineages, indicating a general occurrence of gene flow in the establishment of family groups after 2011. Therefore, to understand the effect of lineage cross events on the future demography of the Hainan gibbon, we performed a population viability analysis (PVA), which combines our genomic results with information about demographic fluctuations and life history characteristics of the Hainan gibbon to parameterize stochastic, individual-based simulations using SLiM (*29*). Our simulations showed that the extinction rate of the gibbon population in the next 100 generations is 5% when all mating involves individuals from different lineages and is 10% when half of the mating is between individuals from different lineages. These are much lower than the estimate without taking into account the cross of two genetic lineages (40%) (Fig. 4E). In the future, a greater potential for population growth and a significantly lower level of inbreeding are also expected with continuous crosses between the two gibbon lineages (all *P* < 0.0001, Wilcoxon signed-rank test; Fig. 4E).

## DISCUSSION

Thanks to recent conservation endeavors, there are a few successful cases of critically endangered species that have rebounded from their population bottlenecks. While evidence is accumulating about threats causing species endangerment, the genomic mechanisms underlying species rebound remain an outstanding question. In this study, we used fecal and historical genomics data to untangle the interplay of multiple evolutionary forces during the mysterious rebound of a critically endangered primate species, the Hainan gibbon. We reveal that the population rebound of this species has been owed to its genetic legacy. This legacy mainly came from a millennial-scale population expansion that had mitigated the negative genetic consequences of the prolonged LGM-associated bottleneck and from the high local genomic recombination that had reduced genetic load and helped maintain functional variation. We further found that recent natural gene flow and hybridization between the two long-separated genetic lineages had promoted the population growth of this most endangered primate species.

Although a few previous studies have attempted to enrich host DNA from fecal samples [e.g., (*19*, *30*)], the wide application of fecal genomics in conservation genetics remains limited due to the perception that its inherent properties (e.g., extremely low DNA amount and high DNA fragmentation) will lead to potential high ADO (*31*) and poorly understood potential effects on downstream genetic analyses. In this study, we present a systematic pipeline that includes strict sampling procedures, effective host DNA enrichment, and adequate data quality control (e.g., assessing and controlling genotyping errors, particularly ADO; fig. S1). Our results demonstrate that the NGS-based fecal genomic approach can overcome these limitations and generate a reliable SNP dataset, characterized by a minimized ADO rate and a sufficient number of usable SNPs (figs. S4 to S8 and Supplementary Text). Our study thus pioneers and demonstrates an applicable approach for conservation genomics, especially for endangered species, where obtaining high-quality genetic samples in the wild is often challenging, marking a substantial leap forward in using noninvasive fecal samples for conservation genomics.

Recent biodiversity loss is thought to largely result from intensified anthropogenic disturbances (*32*). However, numerous recent genomic studies have found that many endangered species’ populations had already started to decline before the LGM [e.g., (*4*)] when humans were still not populated (*8*), implying that species demography must have played an earlier role during these endangerment processes. Our research on the Hainan gibbon, while verifying a prolonged population bottleneck typically observed in many endangered species and associated with the LGM [e.g., (*4*)], reveals a millennial-scale expansion that likely minimized the harmful genetic consequences of this bottleneck. This is different from most endangered species reported before [e.g., (*4*, *29*)], but similar scenarios were found in recent human history, in which millennial population expansions have facilitated selection efficiency, maintained harmful alleles at low frequencies, and also reduced the inbreeding level (*8*, *9*). The inferred population expansion of Hainan gibbons at 5 kya agrees well with the event that savannahs were replaced by closed-canopy rainforest in Southeast Asia (*33*), which may also explain the endemism of many gibbon and rainforest species (e.g., Hainan peacock pheasants; *Polyplectron katsumatae*) in this global biodiversity hotspot (*34*, *35*). Most demographic inference methods, including those used in this study, assume panmictic populations and do not account for the potential influence of historical population structure (*36*). Therefore, the inferred historical fluctuations in *N*_e_ may reflect not only changes in census population size but also signals arising from historical population structure or a combination of both.

Past theoretical studies predicted that endangered species would harbor a high proportion of harmful mutations (genetic load) due to strong genetic drift and reduced selection efficiency resulting from small population size (*6*). However, this is not the case for the Hainan gibbon that has a low genetic load despite the lowest census and effective population sizes among all the studied primates. Our study suggests that high local recombination (genome architecture more generally) in Hainan gibbons may have contributed to alleviating the genetic load due to strong genetic drift during the most serious population decline from 1950 to 2003 (Fig. 2A) by increasing purifying selection efficiency and maintaining important functional variation. In addition, almost all extant small apes including Hainan gibbons are listed as “endangered or critically endangered” by international societies mainly because of their small census sizes or limited range areas (*37*). However, different from great apes and humans, previous studies have shown that small apes featured unusually accelerated rates of chromosomal rearrangements (*34*). Because chromosomal rearrangements can substantially affect the recombination landscape and consequently influence the efficiency selection (*38*), together with a relatively lower genetic load of small apes (figs. S15 and S16 and tables S6 and S7), these phenomena suggest that they might have more potential for recovery from population bottlenecks than great apes.

As group-living animals, most primates have postnatal dispersal to join or establish family groups primarily through mating with unrelated or distantly related individuals (*39*), which could reduce inbreeding risk and alleviate food resources competition and intrasexual breeding competition (*40*). However, few studies have investigated whether these behavioral regulations could help save endangered primates from extinction. Our work found that some Hainan gibbon individuals, facing habitat conversion and forest fragmentation, had successfully colonized new territories by bisexual dispersals during the recent two decades (Fig. 4C) (*16*), resulting in the crosses of the two distinct genetic lineages that had been separated about 450 years ago. This dispersal and hybridization may have contributed to the population’s rebound from the most serious population bottleneck around 2003 (Fig. 2A) and could provide a greater chance for their future population growth (Fig. 4E). Our elucidation of the genetic basis for Hainan gibbon’s rebound highlights the importance of understanding demographic history, genome architecture, and behavioral regulation in the protection of threatened primates.

## MATERIALS AND METHODS

### Sample collection

Fecal samples of the Hainan gibbon were collected from the Bawangling National Nature Reserve, Hainan, China, from February to July 2017, August to October 2020, and July to August 2021, respectively. To cover the current gibbon distribution as much as possible, we surveyed their habitats using a triangulation method and searched fresh fecal samples wherever possible using a spot-site observation method (*15*). Specifically, we tracked the Hainan gibbon family groups by vocalization and observed them until defecation. Fecal samples were randomly collected from each family group. The fresh feces were immediately transferred to a 15-ml sterile microcentrifuge tube (Corning, USA) and promptly stored in liquid nitrogen to prevent DNA degradation. A total of 42 fecal samples was lastly obtained (table S1). We further collected four museum samples: two collected from Wuzhi Mountain in 1960 and stored in the Museum of Institute of Zoology, Guangdong Academy of Sciences, and two collected from Bawangling areas in 1984 and stored in the Museum of Bawangling Sub-Management Bureau of Hainan Rain Forest National Park (data S1). In addition, we collected one blood sample of the yellow-cheeked gibbon (*Nomascus gabriellae*) reared at the Nanning Zoo, China (data S1).

### Individual identification and sex determination

The genomic DNA was extracted from fecal samples using a QIAamp Fast DNA Stool Mini Kit (QIAGEN, German) following the manufacturer’s instructions. DNA yield was estimated using a Qubit 2.0 Fluorometer (Thermo Fisher Scientific, USA). To conduct individual identification, we used the microsatellite system of 10 markers that we have developed (*18*). For each sample, four multiplex group polymerase chain reactions (PCRs) were performed in a 10-μl reaction volume with 1 μl of template DNA, 0.5 μM forward and reverse primers, respectively, 5 μl of QIAGEN Multiplex PCR Master Mix (QIAGEN, Germany), and 0.2 μg/μl of bovine serum albumin. The PCR conditions were set as follows: Group 1: 94°C for 15 min, followed by a touchdown procedure (a total of 35 cycles of 94°C 30 s, *T*_anneal_/120 s, and 72°C/45 s) and a final step of 72°C for 5 min. The *T*_anneal_ was decreased by 0.5°C every second cycle from 65° to 55°C over 20 cycles. Groups 2 to 4: The conditions were the same as that of group 1 except for *T*_anneal_ (58° to 48°C for group 2, 56° to 46°C for group 3, and 53° to 43°C for group 4). Each sample was amplified with three replicates in each multiplex group. PCR products were analyzed using an ABI 3730xl DNA Analyzer (Thermo Fisher Scientific, USA), and genotypes were scored using GeneMarker version 2.2.0 (*41*). The scored genotypes were further processed by Microsatellite Toolkit (*42*) for individual identification (data S2).

The sex of each individual was molecularly determined using a similar reaction system as above mentioned but with different primers: A-UTXTUY_F1 (0.4 μM), A-UTXR1 (0.2 μM), A-UTYR1 (0.2 μM), A-SRY_F1(0.2 μM), and A-SRY_R1 (0.2 μM) primers (*18*). The amplification condition was set as 94°C for 15 min, 40 cycles at 94°C for 30 s, 57°C for 30 s, 72°C for 30 s, and a final extension at 72°C for 5 min. The PCR products were lastly run on a 3% agarose gel with three clean bands being considered to be a male and a single band a female. The final results of individual identification and sex determination are shown in table S2.

### Host DNA enrichment

#### Fecal DNA extraction

We used the QIAamp Fast DNA Stool Mini Kit to extract DNA from each identified individual. Different from the DNA extraction in Individual identification and sex determination section, we optimized the PCR inhibitor removal step in the protocol provided (isolation of DNA from stool for human DNA analysis). Specially, each extract was divided into two subextracts, for which we reduced the amount of feces to 50 mg and increased the InhibitEX buffer volume to 1.5 ml (fig. S2). The two subextracts were then combined into one QIAamp spin column to ensure the DNA yield. For each individual, DNA was extracted several times (average: 9, range from 4 to 19), and the concentration was estimated for each extract using a Qubit 2.0 Fluorometer.

#### Host DNA quantification before enrichment

To estimate the host DNA amount in the fecal DNA extracts, we used a quantitative PCR (qPCR) protocol of Morin *et al.* (*43*). Previous studies showed that using a standard *C*_t_ curve generated from high-quality DNA extracts could increase the determination accuracy of host DNA amount by at least five orders of magnitude (*43*); we therefore extracted DNA from the blood sample of the yellow-cheeked gibbon using a Blood & Cell Culture DNA Midi Kit (QIAGEN, German) to establish the standard curve. We then used Primer3 version 4.1.0 (*44*) to design the qPCR primers (forward, 5′-AACACACAACGTCTTGGAGC-3′, and reverse, 5′-GGGGCCTTTTCATTGTTTTCC-3′) based on the *c-myc* gene sequence obtained from white-cheeked gibbon (Asia_NLE_v1, accession no. GCA_006542625.1). For the qPCR amplification, a 10-μl reaction system contains 1 μl of template, 0.5 μM forward and reverse primers, respectively, and 5 μl of 2X PowerUp SYBR Green Master mix (Thermo Fisher Scientific, USA), and reactions were performed on an Agilent Technologies Stratagene Mx3000P (Agilent, USA) with an initial denaturation of 50°C for 2 min and 95°C for 2 min, followed by 40 cycles of 95°C for 15 s, 59°C for 15 s, 72°C for 1 min, and the last cycle of 95°C for 15 s, 60°C for 1 min, and 95°C for 15 s. The yellow-cheeked gibbon DNA extracts with different concentrations (10, 8, 4, 2, 1, 0.8, 0.4, 0.2, 0.1, 0.08, 0.04, 0.02, and 0.01 ng/μl) were used as standard DNA templates and Hainan gibbon fecal DNA extracts as tested templates. Every standard and tested DNA extracts were run in triplicate with the mean *C*_t_ value calculated. Host DNA amounts were estimated from the slope and *Y* intercept (*Y*_int_) of the trendline from the standard curve, plotted as the log_10_ of the host DNA amounts versus the *C*_t_ values: host DNA amount = $10^{\left[\left(Ct-Y\text{int}\right)/\text{slope}\right]}$. A standard curve was rejected if its correlation coefficient was <0.9.

#### Host DNA enrichment

The fecal DNA extract normally contains a very small proportion of host DNA (e.g., ~1%) (*19*). To overcome this problem, we used an NEB Next Microbiome DNA Enrichment Kit (New England Biolabs, USA) to enrich endogenous DNA from the extracts (*19*). This method uses a methyl-CpG–binding domain (MBD) protein to selectively bind and isolate endogenous DNA with high CpG-methylation density because most exogenous DNA (e.g., bacterial DNA) has low CpG-methylation density. MBD2-Fc–bound magnetic beads were prepared according to the manufacturer’s instructions. For each extract, we adjusted the bead volume according to its host DNA amount (estimated by the above qPCR analysis) and kept each capture reaction volume constant at 120 μl (80 μl of DNA, 20 μl of water, 20 μl of bind/wash buffer). Different from the recommended protocol, we added an extra wash in which samples were resuspended in 100 μl of 1× bind/wash buffer, incubated at room temperature for 3 min with rotation, and eluted in 100 μl of 2 M NaCl. After the host DNA enrichment, qPCR was used again to estimate the proportion of host DNA using the same procedure mentioned above. The enrichment fold was estimated by dividing the preenriched host DNA proportion by the postenriched host DNA proportion (data S3). To meet the requirement of the following sequencing library construction (i.e., ≥50 ng of DNA), multiple enriched DNA extracts from the same individual were pooled together.

### Library construction

Sequencing libraries were constructed using an NEB Next Ultra II DNA Library Prep Kit for Illumina (New England Biolabs, USA) following the manufacturer’s instructions, and index codes were added to attribute sequences. Briefly, the genomic DNA sample was fragmented by sonication to a size of 350 base pairs (bp). Then, DNA fragments were end polished, A tailed, and ligated with the full-length adapter, followed by PCR amplifications with 12 cycles for each sample. The PCR products were purified by the AMPure XP system (Beckman Coulter, USA) to finish the library construction, and the DNA concentration was quantified using a Qubit 2.0 Fluorometer. The constructed libraries were analyzed for size distribution on an Agilent 2100 Bioanalyzer (Agilent, USA) and quantified by real-time PCR (concentration > 2 nM).

### Genome resequencing

The libraries were sequenced using 2 × 150 bp reads on an Illumina HiSeq X Ten platform (Illumina, USA). To determine the amount of raw sequencing data for optimal genome coverage (i.e., the proportion of sequenced bases in the reference genome), we first generated raw sequencing data of 10 or 30 Gb. We then extracted data of given amounts (e.g., 5, 10, 15, 20, 25, and 30 Gb) from the sequenced data and respectively mapped these data against the reference genome of the northern white-cheeked gibbon (*N. leucogenys*), available in the National Center for Biotechnology Information (NCBI) RefSeq database (accession: GCF_006542625.1). We performed read alignment using Burrows-Wheeler Alignment tool (BWA) version 0.7.17 (*45*) with default parameters. We estimated the genome coverages for different amounts of data assuming that the two variables follow a Solow growth model as below

$$y=a-e^{-\mathrm{bx}+c}$$

where *y* is the genome coverage, *x* the data amount, *a* is the maximal genome coverage (a constant), and *b* and *c* are the constants. The three constants (*a*, *b*, and *c*) could be resolved after introducing the estimated genome coverage of different amounts of data into the equation. On the basis of the resolved equation (table S3), we sequenced the genome with an amount that allows the genome coverage to reach the tipping point (fig. S3).

### Effectiveness of host DNA enrichment

The effectiveness of host DNA enrichment for each individual was evaluated by assessing enrichment efficiency, enrichment sensitivity, genome coverage, and library complexity. Enrichment efficiency was calculated as the number of reads mapped to the reference genome of *N. leucogenys* divided by the number of raw reads. Enrichment sensitivity was defined as the proportion of uniquely mapped reads among the total mapped reads. The genome coverage was defined as the proportion of mapped bases in the reference genome. Library complexity was indexed by the duplicate rate. The final results are provided in data S4.

### Data processing and SNP calling

The SNPs were called using the pipeline that we have developed previously (*46*). Briefly, after adapter trimming, reads were aligned to the reference gibbon genome of *N. leucogenys* using BWA with default alignment parameters. Duplicates were removed using Picard. The reads mapped to multiple genomic locations or with a mapping quality score < 20 were excluded using SAMtools version 1.9 (*47*). The variants and genotypes were identified using the GATK (gatk-package-4.1.8.0-local.jar) (*48*). The variants were filtered using the following criteria: variant failed if GQ < 20, QD < 2.0, MQ < 50.0, FS ≥ 40.0, SOR > 5, BaseQRankSum < −3, MQRankSum < −12.5, and ReadPosRankSum < −3.0.

### Estimation of ADO rate, GC content, and randomness of enriched host DNA fragments

The ADO rate in SNP genotypic data was estimated using two methods. The first is the trio experiment that uses the genotypes of an offspring and its parents in detecting ADOs based on Mendelian inheritance theory (*49*). When the parental genotypes are AA and Aa and the offspring is aa, there is an ADO occurrence in the offspring genotype at the locus. Similarly, when the parental genotypes are AA and aa but the offspring’s genotype is AA or aa, it also indicates an ADO at this locus. With the second method, we randomly select three individuals and construct two sequencing libraries for each individual. We calculated the genotype discordances between the sequencing data from the two libraries. This method estimates not only ADO but also other types of random or systematic errors such as false alleles. To determine the optimal SNP depth that minimizes ADO, we calculated ADO, by the Mendelian inheritance and genotype discordance methods described above, across different SNP depths and established the relationship between ADO and SNP depth.

To further assess the impact of ADO rates on downstream genetic analysis, we selected SNP datasets at different sequencing depths and compared results across different ADO rates, ranging from high to low. Three genetic parameters were used as representative metrics to evaluate this effect: genome-wide heterozygosity, inbreeding levels, and genetic load (deleterious mutation and LoF mutation).

To evaluate whether the CpG-based host DNA enrichment method introduces a GC content bias in the resulting sequencing data, we followed the protocol reported previously (*19*, *30*) and used the museum samples of Hainan gibbon as a control. We calculated the GC content for 13 current Hainan gibbon individuals and four museum specimens. We then compared the mean GC content of genomes obtained using the enrichment method with those from museum genomes processed without CpG enrichment.

To check the representative of the sequenced DNA fragments, we examined the distribution of sequencing reads across genomic windows (100 kb). A distribution that a normal distribution would indicate that the reads were mapped randomly to the reference genome. Two individuals were selected as representatives, one with relatively high data quality (A01) and the other with relatively low data quality (A04), based on a comparison of parameters such as mapped reads (199,109,142 for A01 versus 23,024,914 for A04), enrichment efficiency (19.79% for A01 versus 1.71% for A04), and sequencing genome coverage (68.06% for A01 versus 21.65% for A04), among others (please see data S4).

### Data processing and SNP calling of other primate species

To understand the population genetic status of the Hainan gibbon among primate species, we obtained resequencing genomic data of primate species or subspecies with different conservation statuses from GenBank. The selection criteria were listed as follows:

1) For each species, there are at least three individuals with resequencing genomic data.

2) The species whose sampling location information is unknown or whose samples come from a single sampling locality are excluded.

In this study, following the recommendation of Kuderna *et al.* (*50*), we grouped the International Union for Conservation of Nature critically endangered, endangered, and vulnerable species as threatened primates and near threatened and least concerned as unthreatened primates. In the end, we obtained resequencing genomic data of 18 threatened primate species or subspecies and five unthreatened primate species from GenBank (data S6).

The genomic sequencing reads from every species were aligned to their respective reference genomes or reference genomes of their relative species using BWA (data S6). SNP calling was performed using the aforementioned procedure but with varying filtering criteria (data S6).

To eliminate any potential bias caused by the difference in sequencing depth among the Hainan gibbon and other primate species, we downsampled the genomic data of the other species to an average sequencing depth of 10× to be comparable with the Hainan gibbon data for downstream analyses.

### DNA extraction, sequencing, and data processing of museum samples

The DNA extraction, library construction, sequencing, and data processing of museum samples were conducted using the protocol we developed previously (*51*).

#### DNA extraction

We used 25 mg of each skin, cut it into small pieces of size < 1 mm^3^ using sterile scissors, and placed them into a 2.0-ml PCR-clean DNA LoBind tube (Eppendorf, Germany). The sample pieces were rinsed with 70% ethanol (Sigma-Aldrich, USA). The supernatant was removed after centrifugation. This step was repeated three times, and the DNA extraction was performed according to the protocol described in Wang and colleagues (*51*). The sample preparation was performed in a clean room at the Laboratory on Molecular Paleontology in the Institute of Vertebrate Paleontology and Paleoanthropology, Chinese Academy of Sciences, and all experiments were conducted with strict contamination controls.

#### Library construction

Libraries were all treated with uracil-DNA-glycosylase and endonuclease (Endo VIII) to remove characteristic ancient DNA deamination. The libraries were PCR amplified using AccuPrime Pfx DNA polymerase (Life Technologies, USA). Sample-specific indexes were introduced into both the P5 and P7 adapters during library amplification to make it possible to distinguish samples from the new libraries from any other library. Library concentrations were determined using a NanoDrop 2000 spectrophotometer and a DNA-1000 chip on an Agilent Bioanalyzer 2100.

#### Sequencing, data processing, and SNP calling

The libraries were sequenced using the same sequencing platform as mentioned above. Adapters were trimmed, and paired-end reads were merged into one single read (minimum overlap of 11 base pairs). The merged reads were aligned against the reference genome of *N. leucogenys* using BWA version 0.6.1. All duplicates (reads mapped to exactly the same position of the genome) were removed with bam-rmdup (https://github.com/mpieva/biohazard-tools). The SNP calling for museum samples was conducted using the same pipeline as mentioned above (please see Data processing and SNP calling section). To eliminate any potential bias caused by the difference in sequencing depth among the current samples and museum samples of Hainan gibbons, 10× SNPs from museum samples were used in the downstream analysis.

### Pedigree construction

On the basis of the genomic SNPs identified, we inferred the genealogical relationships among gibbon individuals using RELPAIR version 2.0.1 (*52*), which adopts a likelihood method to infer the most likely relationship of any two individuals from a total of eight candidate relationships (monozygotic twins, full siblings, parent/offspring, half-siblings, grandparent/grandchild, avuncular, first cousins, and unrelated). To mitigate the potential impacts of selection and excessive missing data, we kept the SNPs that meet the following criteria: (i) no significant deviation from Hardy-Weinberg equilibrium, (ii) ≤50% missing alleles, (iii) the read count ratio of major allele divided by minor allele for each heterozygote ≥ 0.5 and ≤ 2, and (iv) MAF (minor allele frequency) > 0.3. To exclude the linkage effects, we further divided the genome into 2748 windows (1 M) and adopted a 100-bootstrap strategy to remove closely linked markers. For each bootstrap, we randomly selected one SNP from each window to yield a total of 2748 SNP loci for relatedness estimation. A relatedness estimate was accepted when supported by at least 90 bootstrap analyses.

### Census population sizes of Hainan gibbons

We searched the literature on the population size of Hainan gibbons from 1950 to 2021 and characterized the population size changes since 1950, when its population started to decline rapidly.

### Estimation of effective population size and heterozygosity

To estimate the *N*_e_ and heterozygosity of the Hainan gibbon population during the decline stage, we used SNP genotype data from individuals alive during that period (*N* = 13).

For *N*_e_ estimation, we selected the individuals from the same generation (*N* = 9) to avoid the Wahlund effect (*53*). We used the LD method as implemented in NeEstimator version 2.1 (*54*). When using the LD-based method, it is more accurate to estimate the number of effective breeders, *N*_b_. As the closest surrogate of *N*_e_, *N*_b_ provides an approximate measure for interpreting inbreeding and genetic drift, and it is more sensitive than *N*_e_ for monitoring demographic changes (e.g., population decline) in a population with overlapping generations (*55*). This information is crucial for understanding the genetic status of the Hainan gibbon in 2003, following an extreme bottleneck event.

The default parameters were used to estimate the *N*_e_ for Hainan gibbons and other primate species from their SNP genotype data. Before estimation, all SNP datasets were screened with the following criteria: (i) no significant deviation from Hardy-Weinberg equilibrium, (ii) no missing data, (iii) autosomal SNPs, and (iv) MAF > 0.1. The heterozygosity of each primate individual was calculated as the number of heterozygous sites divided by the effective length of the reference genome.

### Evaluation of inbreeding level and genetic load

The inbreeding level *F*_E_ of each primate species was estimated using FEstim (*20*), which calculates the probability of identity by descent for two alleles in an individual. *F*_E_ has been shown to outperform the proportion of the genome in ROH (*F*_ROH_) when relatively few SNPs are available, providing higher precision and lower bias (*56*), making it suitable for our fecal DNA dataset. Specifically, we chose the loci with the following criteria: (i) from autosomes, (ii) no significant deviation from Hardy-Weinberg equilibrium, (iii) loci detected in more than 80% of individuals, and (iv) MAF > 0.1.

The estimation of genetic load followed the protocol described in Feng *et al.* (*57*). The genetic load of each individual from each primate species was estimated on the basis of missense mutations and LoF mutations. As there is currently no reference genome available for the Hainan gibbon, we followed the common practice in studies of other wild animals (*58*) and adopted the reference genome and annotation of its closely related species, the northern white-cheeked gibbon, obtained from the NCBI RefSeq database (accession: GCF_006542625.1; Annotation Release 103). Missense mutations were classified into two categories according to the Grantham score (ranging from 5 to 215) with the deleterious mutations scoring ≥150 and benign <150. LoF mutations were the variants leading to stop codons or splice site disruptions on gene sequences.

The homozygous genotypes of the major alleles were considered as the ancestral state.

The genetic load in homozygote state was estimated using the equation below

$$\begin{array}{c} \text{Genetic load for LOF mutations and deleterious mutations}=\\ \frac{2\times \text{the number of derived homozygous sites}}{\left(2\times \text{the number of derived homozygous sites}+\text{the number of heterozygous sites}\right)} \end{array}$$

A larger value means a stronger genetic load.

### Demographic history reconstruction of Hainan gibbons

We reconstructed the demographic history of Hainan gibbons using four methods with different algorithms.

#### Pairwise sequentially Markovian coalescent modeling

We used the pairwise sequentially Markovian coalescent (PSMC) (*59*) to reconstruct the ancient demographic history of Hainan gibbons over the past 1 Ma. Following the method recommended by Liu and Hansen (*60*), we pooled all autosome sequences of the studied Hainan gibbon individuals. We identified homozygous reference genotypes and missing data that we excluded. We screened SNPs with sequencing depth ≥ 10, sequence quality (*Q* score) ≥ 20, and the read count ratio of major allele divided by minor allele for each heterozygote ≥ 0.5 and ≤ 2 to generate 71 “chromosomes” through referring to the white-cheeked gibbon reference genome. The sequences were then divided into nonoverlapping 100-bp bins, with a bin being scored as heterozygous if a heterozygote exists or as homozygous otherwise. Population histories were inferred by PSMC using parameters “-N25 -t15 -r5 -p 64*1”, and 200 bootstrapping samples were used to obtain the CI. The mutation rate was set to be 1 × 10^–8^ per site per generation, and the generation time was set to 10 years (*34*).

#### Stairway Plot

We used Stairway Plot 2 version 2.1.1 (*61*) to reconstruct the more recent demographic history of Hainan gibbons (50 to 5 kya). To obtain putatively neutral sites, we excluded SNP loci in coding regions and those identified to be under balancing selection (*23*) (please see Balancing selection analysis section). SNP loci with the read count ratio of major allele divided by minor allele for each heterozygote ≥ 0.5 and ≤ 2 were included in the analysis. The folded SFS (site frequency spectrum) was calculated using easySFS version 0.0.1 (https://github.com/isaacovercast/easySFS). Stairway Plot 2 was run with the settings nrand 4 9 14 18, pct_training 0.67, mu 1e-8, and year_per_generation 10.

#### PopSizeABC

To reconstruct the demographic history of Hainan gibbons from 5 kya to the present, we ran an approximate Bayesian computation approach named PopSizeABC version 1.2 (*62*). The SNP data processing was the same as that in the Stairway Plot analysis. The analysis parameters were set as follows: 5000 replications of simulation with an nb_seg (number of independent segments in each dataset) of 25, an *L* (size of each segment, in base pairs) value of 6,000,000; mac [minor allele count threshold for allele frequency spectrum (AFS) and identity by state (IBS) statistics computation] of 0, and mac_ld (minor allele count threshold for LD statistics computation) of 6.

#### GONE

To reconstruct more recent demographic histories of the Hainan gibbon lineages detected in our study, we adopted GONE (*63*) that infers the recent past effective population size trajectory (e.g., ≤100 generations) by modeling the observed LD between pairs of SNPs with different recombination rates. Considering that the GONE analysis might be influenced by the potential existing population structure (e.g., metapopulation composed of two subpopulations), we first performed the population structure analysis (please see Population structure analysis section) and identified two distinct lineages (lineages A and B; Fig. 4, A and B). Therefore, in our study, we generated an SNP dataset for each lineage. SNPs were screened according to the following criteria: autosomal, present in all individuals, MAF > 0.1, no significant deviation from Hardy-Weinberg equilibrium, and neighboring SNPs spaced over 50 kb. A total of 100 independent replicates was run to obtain the CI of the estimated *N*_e_, with each replicate having 10,000 randomly selected SNPs.

### Accumulation of inbreeding and genetic load

The analysis was conducted using a forward-in-time simulation with a non–Wright-Fisher model in SLiM version 3.3.2 (*64*) according to Robinson *et al.* (*29*). Each simulated individual has a diploid ~28-Mb coding region, consisting of 19,197 “genes” distributed on 25 chromosomes as annotated in the *N. leucogenys* genome. Each gene is represented by a contiguous 1458-bp sequence that accumulates mutations at a rate of 1 × 10^–8^ per nucleotide per generation. In our study, because of the lack of mutation rate data from Hainan gibbon pedigrees, we performed a simulation using the human mutation rate (~1 × 10^−9^ per site/year) (*34*). Because of the lack of accurate recombination estimates for the Hainan gibbon, we used the human recombination rate (1 × 10^−8^ per site per generation) in our SLiM model (*65*). The noncoding region between two genes was set at a length of 100 kb, resulting in a recombination rate of 1 × 10^−3^ between genes. Recombination within a gene is not allowed, and recombination between chromosomes is free. In our simulation, 30% of the mutations were assumed to be neutral (*s* = 0), and the remaining 70% were deleterious with selection coefficients drawn from a gamma distribution of fitness effects inferred from a large sample of humans (*66*). Given that deleterious alleles tend to be recessive (*67*), different dominance coefficients for mutants of different deleterious effects (*h* = 0 when *s* < −0.1, *h* = 0.01 when −0.1 < *s* < −0.01, *h* = 0.1 when −0.001 > *s* ≥ −0.01, and *h* = 0.4 when *s* ≥ −0.001) were used in the simulations.

For the gibbon, the mating system is polygynous, usually with one breeding male and two breeding females in each family group. Every breeding year, there are reproduction and viability selection periods (*29*). Our previous study found that a female gibbon started to reproduce at 5 to 8 years old, and each successful copulation produces one offspring (*68*). The breeding interval for females is 2 years, and the female looks after her newborn for 1.5 years. In contrast, the male breeds with one female for the first year and another female in the next year (*68*). Therefore, in our simulations, we set that every breeding male (≥7 years) has two fixed female mates (age ≥ 7 years). If one of the two female mates dies, then the male will have a new mate. In the beginning, the male only mates with a female who has not bred in the previous year. Following reproduction, the viability selection occurs, in which each individual survives with a probability determined by its absolute fitness multiplied by any scaling factors for age or density dependence. To realize the age-specific mortality rates, fitness is rescaled by a factor of 0.1 for newborns, 0.05 for individuals aged 1 to 3, 0.03 for individuals aged 3 to 7, 0.1 for individuals aged 7 to 8, 0.05 for individuals aged 8 to 30, 0.25 for individuals aged 31 to 35, and 0.5 for individuals aged 36 to 40 (*69*). These rescaling factors are multiplied by the absolute fitness of each individual, which also varies as a function of age.

We simulated the inbreeding process and the accumulation of genetic load in a population under two different demographic scenarios: the one inferred for the Hainan gibbon in the present study and a classical island population history (a continuous linear decline from historical to present). The first model comprises sequential events: (i) Burn-in under ancestral conditions: The simulation began with an ancestral carrying capacity (*K*) of 10,000 for 50,000 years for burn-in process. (ii) A population bottleneck preceding the LGM: Following the burn-in, the population underwent a bottleneck representing an ancient decline, with *K* reduced to 500 for 20,000 years. (iii) A subsequent population expansion: The population then expanded over the course of 15,000 years, reaching a peak *K* of 20,000. (iv) A gradual population decline: After the expansion, the effective population size decreased again, with *K* reduced to 3000, lasting until approximately 450 years ago. (v) A recent population split: Around 450 years ago, the population split into two subpopulations (lineages A and B). Since the split, the effective population sizes of both lineages have continued to decline, with *K* reduced to 6 for lineage A and to 5 for lineage B. The second model has the same setting, except for the absence of the expansion phase (event 3).

To estimate the inbreeding level for each simulated generation, we calculated the proportion of *F*_ROH_ with different lengths (≥500 kb and 1 Mb). *F*_ROH_ is calculated using the following formula (*70*)

$$F_{\text{ROH}}=\frac{\sum_{i}\text{length}\left({\text{ROH}}_{i}\right)}{L}$$

Where *i* is the number of ROH identified for each individual in kilobases, length (ROH*_i_*) is the length of each individual ROH segment in kilobases, and *L* is the total length of the genome. To quantify the genetic load in each simulated generation, we first grouped deleterious mutations as very strong (*s* ≤ −0.1), strongly (−0.1 < *s* < −0.01), moderately (−0.01 < *s* < −0.001), and weakly deleterious (*s* ≥ −0.001). The genetic load for each type of deleterious mutation in homozygous state (realized load, which lowers the existing population’s fitness) was lastly calculated using the method mentioned above (“Evaluation of inbreeding level and genetic load”). To characterize the influence of recent historical expansion on genetic load, we also calculated the genetic load for each type of deleterious mutation in heterozygous state (masked load) (*71*), which would reduce the fitness of the future populations through inbreeding and genetic drift (please see the section of Accumulation of genetic load in heterozygous state in Supplementary Text).

### Estimation of recombination pattern across the genome

The GC content at the fourfold degenerated site of protein-coding genes (hereafter GC4), a proxy for the local recombination rate in primates (*22*), was computed using an in-house PERL script for each primate species. SNP loci with the read count ratio of major allele divided by minor allele for each heterozygote ≥ 0.5 and ≤ 2 were used for analysis. The genes with the top (bottom) 50% high GC4 content were considered as the ones with high (low) recombination (*72*).

### Estimation of recombination rate based on the reconstructed gibbon pedigree

We estimated the recombination rate within a specific region, such as a gene region using pedigree information. For each pair of neighboring phased alleles, a crossover event was considered to occur if one allele was inherited from one parent and the other allele from the other parent (e.g., *AB* from one parent and *ab* from the other, with the offspring inheriting *Ab* or *aB*). The recombination rate for the region was then calculated by dividing the number of crossover events by the total number of neighboring phased allele pairs in that region.

### Balancing selection analysis

To identify signatures of balancing selection in the Hainan gibbon population, we first calculated the folded SFS based on the SNP data. Then we used BetaScan (*23*) to calculate the allele frequency correlation summary statistic, ß for each locus. The ß statistic can detect clusters of intermediate-frequency polymorphisms surrounding a central balanced variant. The loci with top 5% ß values were considered to be subjected to balancing selection.

### Population structure analysis

To investigate whether the genetic structure of the Hainan gibbon existed before the recovery stage, we used the individuals assigned to the decline stage (Census population size changes and individuals’ assignment in Supplementary Text) in an unsupervised population structure analysis from SNP genotype data. We inferred the population structure of Hainan gibbons using a likelihood approach implemented in ADMIXTURE version 1.3.0 (*73*). Before the analysis, we used VCFtools to prune our SNPs to retain biallelic ones with minor allele frequencies ≥ 0.05 (--maf 0.05) and with missing data less than 40% (--max-missing 0.4). We also thinned the data to keep SNPs separated by at least 1 kb to reduce the effects of tight physical linkage. We used PLINK v.1.90 (*74*) to convert the data to VCF format and ran ADMIXTURE with *K* = 1 to 4. A *K* with the lowest cross-validation error value was considered the best. Using the same dataset, we also performed a PCA (*75*) and reconstructed a neighbor-joining tree based on pairwise genetic distances (*76*).

### Divergence time estimation between the two genetic lineages

We amplified the mitochondrial DNA control region of the studied Hainan gibbon individuals using the primers of L16007 and H00651 (*77*). The protocol of PCR amplification was derived from our previous study (*77*).

On the basis of our constructed pedigree of the Hainan gibbon, we first excluded three offspring (E01, C07, and C21), which were born after the establishment of group C (Fig. 4C). We then used a CoalHMM version 1.0 (*28*) to infer the divergent time between the genomes of each two individuals from the two lineages, respectively. The genome of each Hainan gibbon individual was reconstructed, referring to the *N. leucogenys* genome and its SNPs as follows: (i) If there exists a homozygous SNP in a Hainan gibbon individual, the corresponding locus in the reference genome is replaced by the Hainan gibbon SNP; (ii) If there exists a heterozygous SNP in the Hainan gibbon individual, the corresponding locus in the reference genome is removed; and (iii) If a locus is inferred to be the same as that of the reference genome, it is kept. We then aligned all the reconstructed genome sequences of Hainan gibbon individuals, divided the aligned sequences into 1-Mb segments, and used the prepare-alignments.py script to generate an input file for CoalHMM. An isolation model implemented in CoalHMM was used for the divergence time estimation with parameters “--Ne 10000 --recomb 0.6 --mu 1E-09 --g 10.”

### Gene flow estimation between the two lineages

On the basis of gibbon population genomic data, we used a coalescent algorithm fastsimcoal2 version 2.6 (*78*) to test the gene flow between lineage A and lineage B under different evolutionary scenarios. First, the autosomal nongenic SNPs with MAF greater than 0.05 and depth above five were used. We used easySFS (*79*) to convert this SNP dataset to an SFS as an input file for fastsimcoal2. Second, we estimated whether and when gene flow occurs between the two lineages under six scenarios: no gene flow, constant gene flow, recent gene flow, early gene flow, high early gene flow and low recent gene flow, and low early gene flow and high recent gene flow (fig. S24). Early gene flow occurred before the population size contraction of lineages A and B (200 to 90 years ago), and recent gene flow occurred after the population size contraction of lineages A and B. The high gene flow (*m1*) means gene flow rate of 0.001 to 0.01, and the low gene flow means 1 × 10^−5^ to 1 × 10^−3^ (*m2*). We tried different settings to optimize the modeling, and we calculated the Akaike information criterion (AIC) values for every model with the one having the smallest AIC being considered the best. Last, to reduce the linkage effects, we split the SNP data into 100 blocks and produced 100 replicate datasets by bootstrapping, with each dataset having one SNP randomly drawn from each of the 100 blocks. We ran the parameter estimation under the best model for 100 times with each of the 100 bootstrapped SFS data. The setting “-n 10000 -L 40 -C 20” was used, and the 95% CI was estimated from the results of the 100 bootstrapping datasets.

### Heterozygosity estimation of hybrid offspring between the two lineages

To investigate the consequence of the cross between lineages A and B, we first compared the expected heterozygosity [*H*_e_ = 2*p*(1 – *p*), *p* is the frequency of the major allele at a locus] of family group C with that of family groups A and B. We then compared the genomic heterozygosity between the hybrid offspring and their parents. For hybrid offspring identification, we used all Hainan gibbon individuals including offspring after the establishment of group C to perform the structure analysis using the same method mentioned above (Population structure analysis section). According to our pedigree result (Fig. 4C and fig. S11) and structure analysis (fig. S26), we found that individual C21 is a hybrid offspring, its father C19 is from lineage A, and its mother C08 is from lineage B. We then compared the heterozygosity between the hybrid offspring and its parents. The heterozygosity distribution along the whole genome for these individuals was estimated using a sliding-window approach (1-Mb size). We also compared the heterozygosity of the hybrid offspring with that of the museum samples from Wuzhi Mountain collected in 1960. Last, we compared the heterozygosity distribution of the offspring from the same lineage (B10 and B11) and the hybrid offspring (C21) from different lineages.

### Contribution of gibbon lineage cross to future population growth predicted by the PVA

The demographic history used in PVA was inferred from empirical data in our study (Fig. 3A) (*29*). During modeling, we simulated future population growth (100 generations, *K* = 2000) under three demographic scenarios: All matings are between individuals from different lineages, half of the matings are between individuals from different lineages, and all individuals mate randomly. Other parameters (e.g., genomic and reproduction parameters) were set as described in the section (“Accumulation of inbreeding and genetic load”). Because hybridization can potentially save a small population from extinction by increasing their population size and reducing inbreeding depression (*10*), we, therefore, focused on the estimation of extinction rate, population growth rate, and inbreeding level (ROH; 500 kb) in our simulations and compared the estimates under the three demographic scenarios in the next 100 generations.

Because we focus on examining the contribution of lineage cross on potential population growth, other processes that maybe also related to the future demography of the Hainan gibbon (e.g., millennial-scale population expansions and local recombination variation) were kept the same during the PVA simulations.

### Statistical analysis

All statistical analyses were performed using R version 4.0.1 (*80*). The data normality was checked using the Shapiro-Wilk normality test. If the data follow a normal distribution, then the significance levels were tested using the Student’s *t* test (two-sided). Otherwise, the Wilcoxon rank sum test (two-sided) was applied. For pairwise testing, the statistical significance was tested using the paired samples *t* test if the data follow a normal distribution. Otherwise, the Wilcoxon signed-rank test was applied. The coefficient of determination (*R*^2^) was used to evaluate the linear relationship, and the significance level was calculated using an *F* test with the *P* ≤ 0.05.

### Ethics oversight

All laboratory experiment procedures were under the guidance of the Ethics Committee of the Institute of Zoology, Chinese Academy of Sciences. The collection and processing of fecal and blood samples in this study were conducted in accordance with the guidelines of the Institutional Animal Care and Use Committee of the Institute of Zoology, Chinese Academy of Sciences (IOZ-IACUC-2021-109).

## Supplementary Materials

### The PDF file includes:

Supplementary Text Figs. S1 to S27 Tables S1 to S8 Legends for data S1 to S12 References
