# Supplementary material for: Genetic legacy and recent cross of two ancient lineages underlie the rebound of the world’s rarest primate
Source: Sci Adv. 2026 Feb 11;12(7):eadw3298. doi: 10.1126/sciadv.adw3298 (PMC12893321; doi:10.1126/sciadv.adw3298)
Supplement: Supplementary file 1 — Supplementary Text Figs. S1 to S27 Tables S1 to S8 Legends for data S1 to S12 References [file sciadv.adw3298_sm.pdf]

Science Advances
AAAS

Supplementary Materials for

Genetic legacy and recent cross of two ancient lineages underlie the rebound of the world's rarest primate

Xian Hou *et al.*

Corresponding author: Jiang Zhou, zhoujiang@ioz.ac.cn; Xiangjiang Zhan, zhanxj@ioz.ac.cn

Sci. Adv. **12**, eadw3298 (2026)
DOI: 10.1126/sciadv.adw3298

The PDF file includes:

Supplementary Text
Figs. S1 to S27
Tables S1 to S8
Legends for data S1 to S12
References

Other Supplementary Material for this manuscript includes the following:

Data S1 to S12

Supplementary Text

Optimal Sequencing Depth Selection for Downstream SNP-Based Analyses

To identify the optimal SNP dataset for downstream analyses, we compared genome-wide heterozygosity, inbreeding coefficients, and genetic load (LoF and deleterious mutations) across sequencing depth thresholds of 6×, 10×, 15×, and 20×. The 6× dataset showed higher ADO rate (Mendelian inconsistency: 1.95% and genotype discordance: 11.54%) compared to 10× (0.95%; 3.15%), 15× (0.6%; 1.5%), and 20× (0.4%; 0.5%), with the latter three falling within recommended coverage ranges for reliable genotype calling in low-coverage or degraded DNA studies (81).

Across these depth thresholds, genome-wide heterozygosity and inbreeding coefficients did not differ significantly between datasets (figs. S4 and 5), and genetic load estimates were comparable among 10×, 15×, and 20×, but not 6× (figs. S6 and 7).

Although the higher depths (15× and 20×) produced estimates comparable to 10× across the three analyses, the sharp reduction in usable SNPs at greater depths led to poorer performance. For heterozygosity, they showed greater variance (fig. S4). For inbreeding coefficients, the assessment at the 20× threshold suffered the limited data, with only 0.69 SNPs/Mb shared across individuals compared with 91 SNPs/Mb at 10× (fig. S8). For genetic load, higher sequencing depths resulted in markedly fewer usable loci (10×: 91 SNPs/Mb; 15×: 53 SNPs/Mb; 20×: 29 SNPs/Mb) and reduced sample sizes passing quality filters (figs. S6 and 7), decreasing from 15 individuals at 10× to 8 at 20× for LoF variants, and to 11 at 20× for deleterious mutations.

Taken together, the 10× SNP dataset is reliable and suitable for downstream genetic analyses.

Pedigree construction

The pedigree of the Hainan gibbon is shown in fig. S11. In our study, there are three pairs of individuals (E02 and B11, A01 and C06, A01, and C19) whose relatedness was supported 50 times for each of the two relationships (e.g., full siblings: 50 times *vs.* parent/off-spring: 50 times). We determined their relatedness listed below.

E02 and B11 were inferred as full siblings or parent/offspring by RELPAIR. Because B11 and B10 were inferred as the breeding parents in Group B and E02 as the offspring of B10 and the full-sib with the offspring (D02, B09, and B07) of B11 and B10 (fig. S11), we thus considered the relationship between E02 and B11 as parent/offspring.

A01 and C06 were inferred as parent/offspring or grandparent/grandchild by RELPAIR. A01 was inferred as the breeding female in Group A, and its offspring A02 and C14 were inferred as the full-sib with C06 (fig. S11). In addition, the RELPAIR is better in inferring first-degree relationships (e.g., parent/offspring) than second or third-degree relationships (e.g., grandparent/grandchild) (82, 83). Therefore, we considered the relationship between A01 and C06 as parent/offspring.

A01 and C19 were inferred as parent/offspring or full siblings by RELPAIR. However, A01 was inferred as the breeding female in Group A. If A01 is the mother of C19, C19 should be full-sib with other offspring (C14, 06, and A02). Otherwise, if A01 is the full sibling of C19, the relationship between C19 and C14, C06 and A02 should be inferred as second-degree relationship or unrelated. In the study, C09 was inferred as the second-degree relationship of C14, C06, and A02 by RELPAIR. Therefore, we considered A01 and C19 as full sib.

Census population size changes and individuals' assignment

We have found a total of 12 papers that have reported the census population sizes of the Hainan gibbon (15, 16, 68, 69, 84-91). From them, we found that the recent demography of the Hainan gibbon comprises two typical stages (Fig. 2A): 1) a rapid population decline from 1950 to 2003, during which the population showed an exponential decline ($R^2 = 0.90$, $p < 0.01$; fig. S12); 2) a growth stage from 2003 to 2021. Our analysis showed that the growth stage fits well with the logistic growth model, that is, the early establishment stage ($R^2 = 0.94$, $p < 0.0001$, fig. S12), which is consistent with the conclusion of a previous study (84).

Accumulation of genetic load in heterozygous state

For the genetic load associated with deleterious mutations in heterozygous state (masked load) of Hainan gibbons, we found that, along with the increase in population size after the LGM, the level of genetic load increased (except for weakly deleterious mutations) and was significantly higher than expected under a classical island population history (figs. S19). With the decrease of population size after 5 kya, the level of genetic load decreased and finally higher than expected under the scenario of a classical island population history (figs. S19). A similar pattern was reported in the previous human study (8). Population growth increases the number of segregating deleterious sites, thus increasing the genetic load in heterozygous state. In contrast, when the population declines, the number of segregating deleterious sites decreases, leading to a lower genetic load in heterozygous state. Furthermore, deleterious mutations exist in the population more easily as homozygous state during population decline, leading to an increased genetic load in homozygous state (realized load) (Fig. 3B).

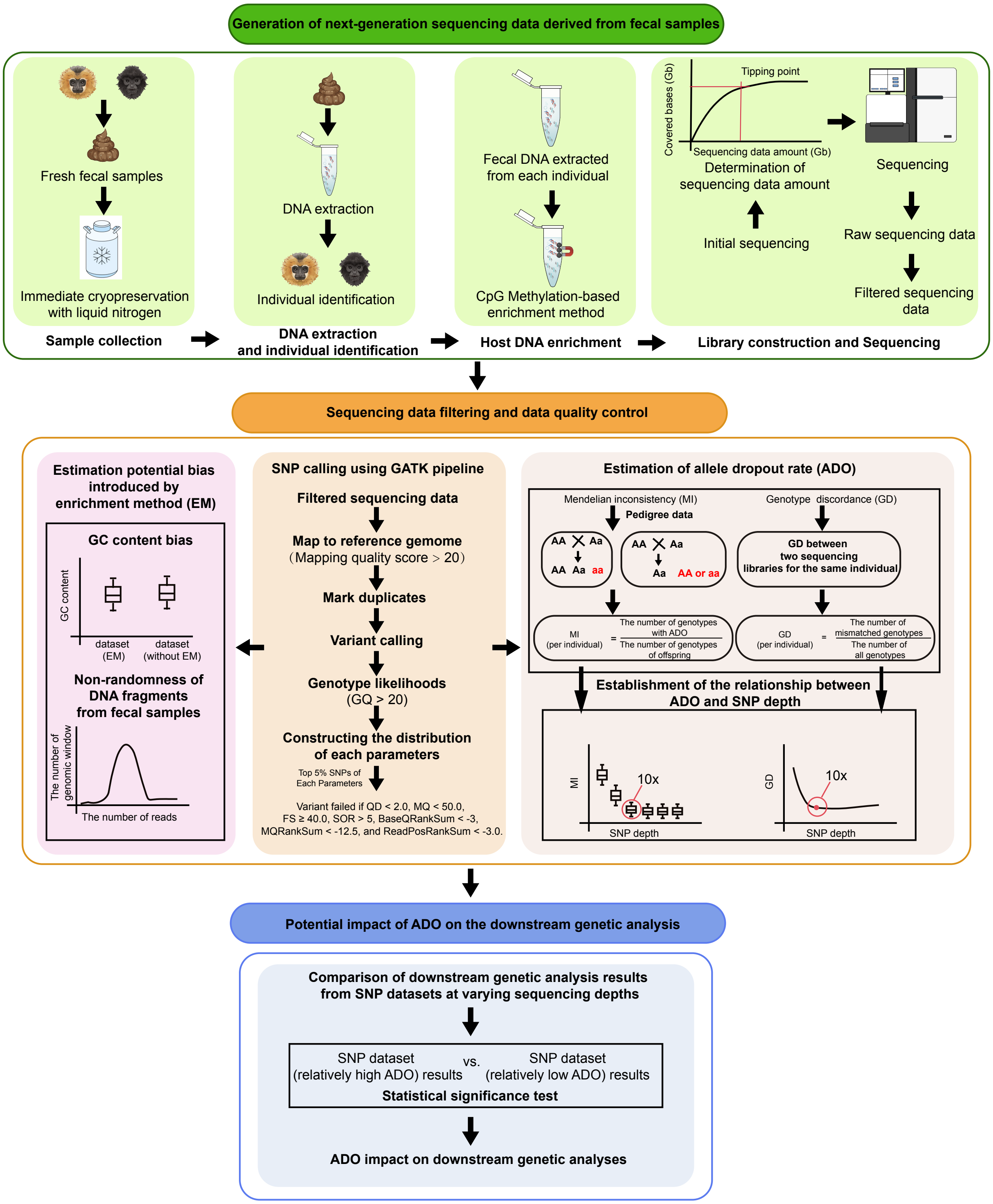

Fig. S1. The systematic pipeline for the generation of fecal genomics data, data quality assessment, genotyping error estimation, and evaluation of ADO effects on downstream analyses.

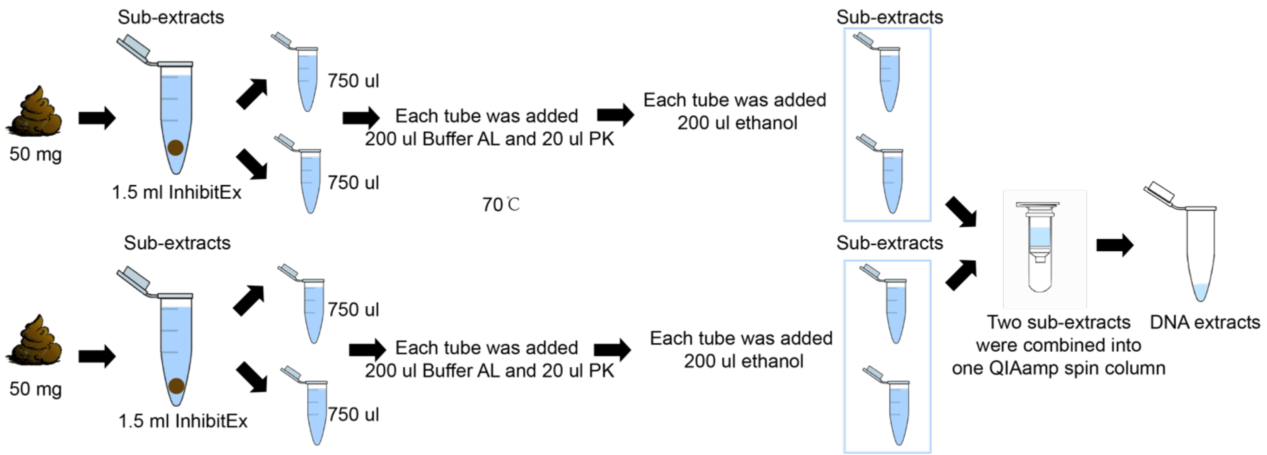

Fig. S2. The flowchart of gibbon fecal DNA extraction. PK denotes the proteinase K.

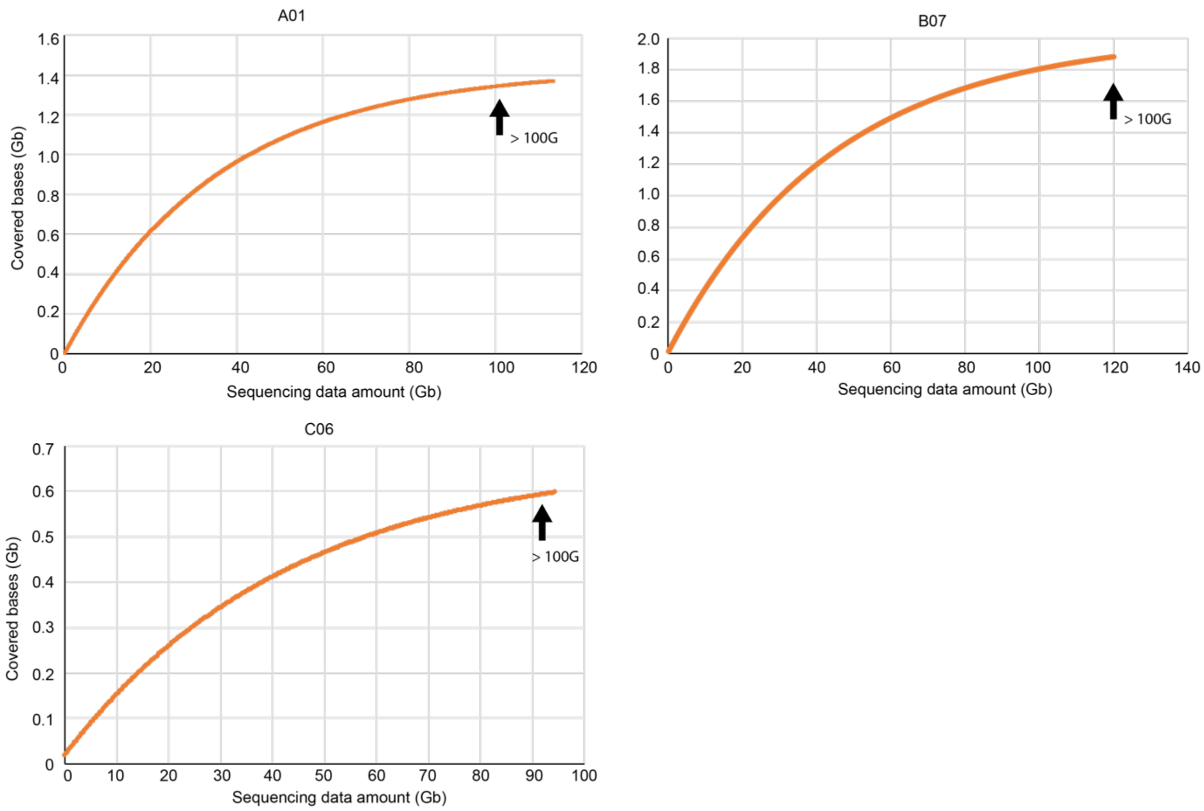

Fig. S3. Examples of determination of sequencing data amount for each individual according to the Solow growth modeling (details in Materials and Methods and table S3).

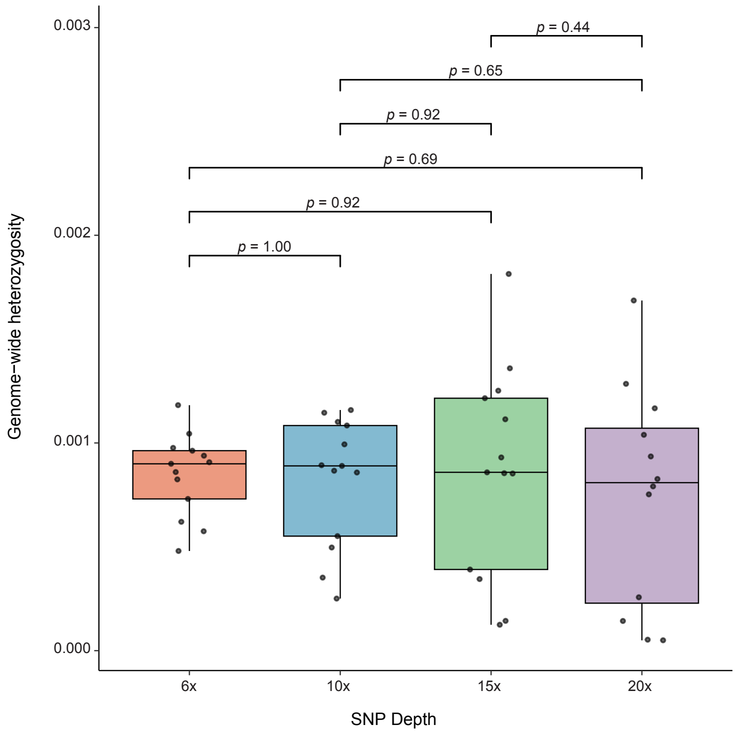

Fig. S4. Comparison of genome-wide heterozygosity estimates based on different SNP depth thresholds. *Wilcoxon rank-sum* tests were applied.

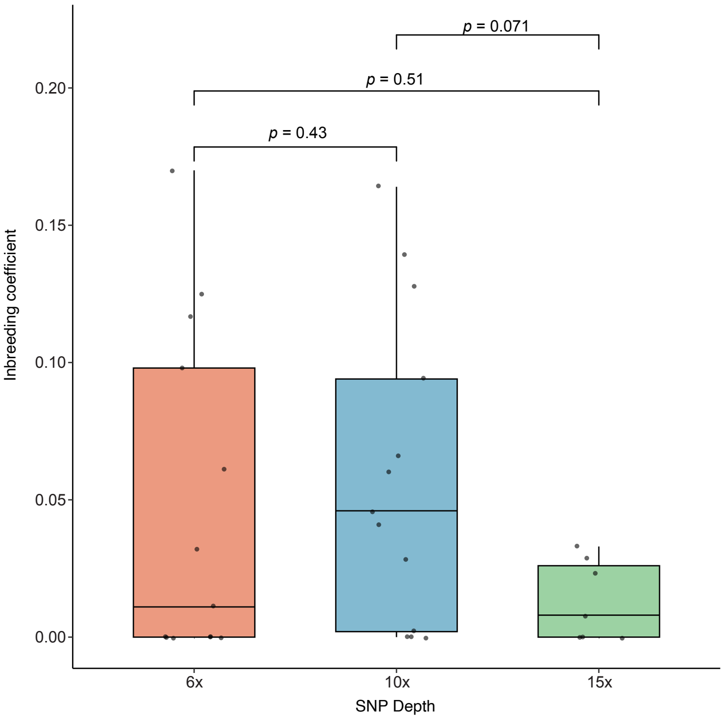

Fig. S5. Comparison of Inbreeding coefficient estimates based on different SNP depth thresholds. *Wilcoxon rank-sum* tests were applied.

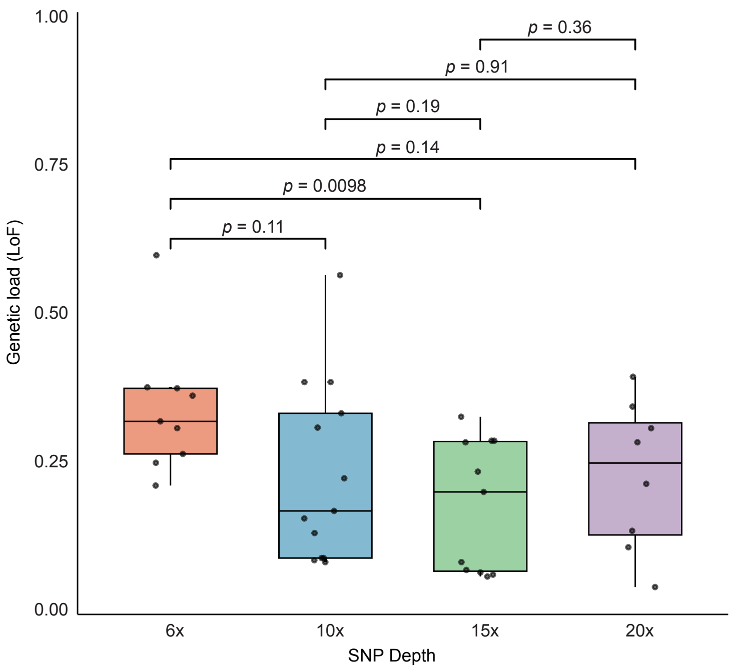

Fig. S6. Comparison of genetic load of LoF estimates based on different SNP depth thresholds. *Wilcoxon rank-sum* tests were applied.

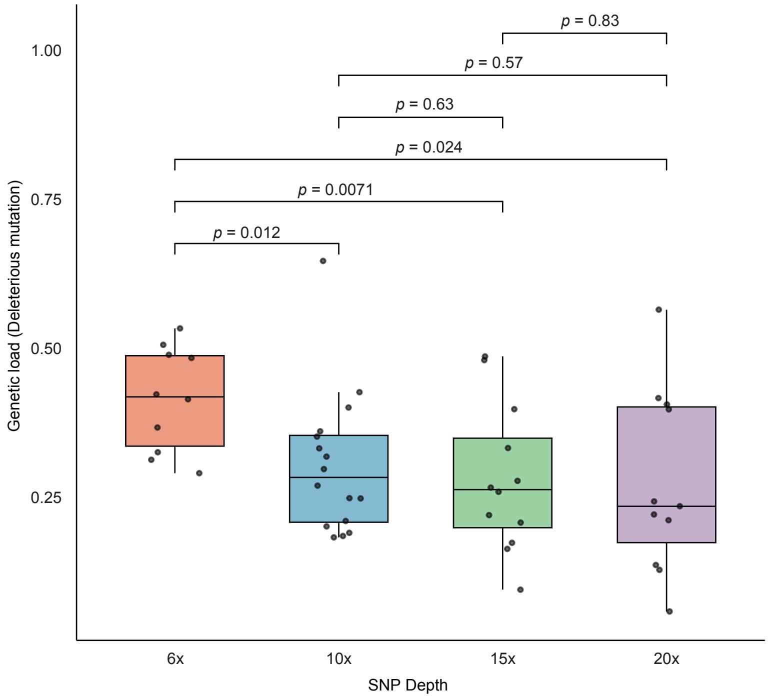

Fig. S7. Comparison of genetic load with deleterious mutations estimates based on different SNP depth thresholds. *Wilcoxon rank-sum* tests were applied.

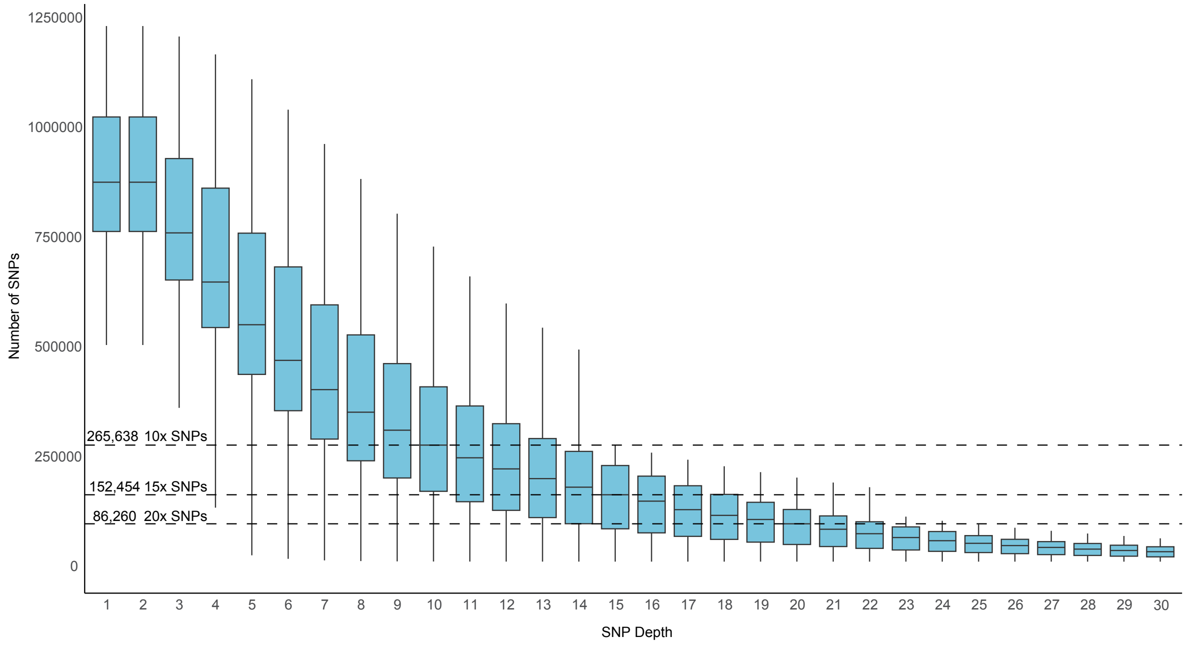

Fig. S8. SNP counts across sequencing depth thresholds. The box plots show the middle bar, upper bound, and lower bound representing the median, the third quartile and the first quartile, respectively; whiskers extend to 1.5 × the interquartile range.

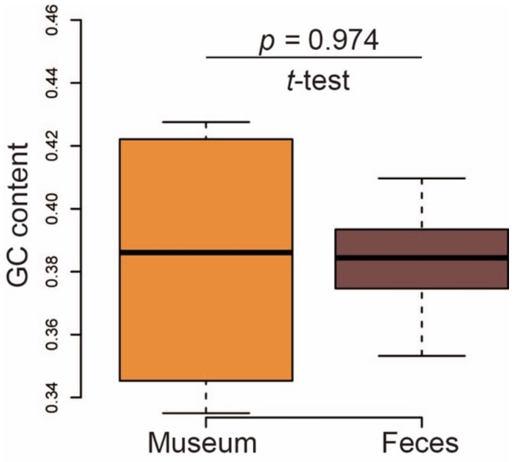

Fig. S9. Comparison of the mean GC content between the gibbon genome sequences obtained from fecal samples and those from museum specimens. The gibbon genome sequences from fecal samples were obtained using a CpG-enrichment-based method and the genome sequences from museum specimens were obtained without using any enrichment method.

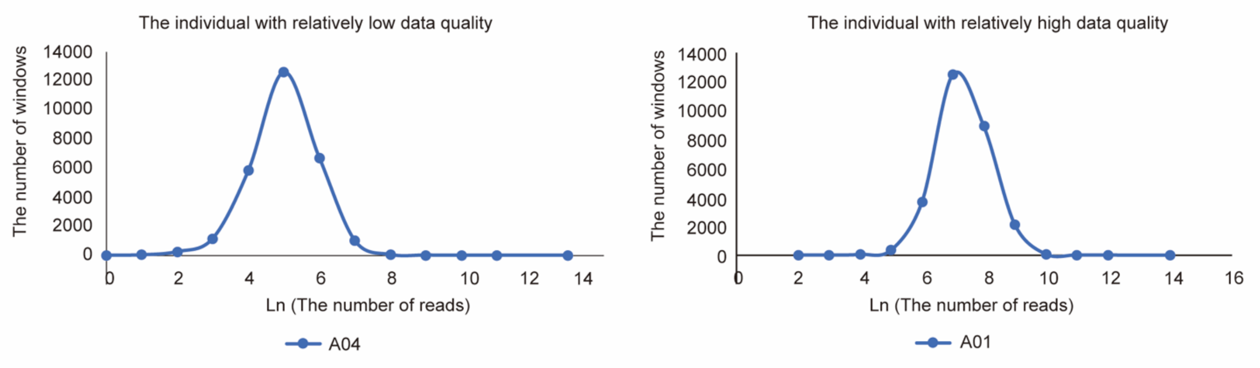

Fig. S10. The randomness of read mapping. Two individuals were selected as representatives, one with relatively high data quality (A01) and the other with relatively low data quality (A04), based on a comparison of parameters such as mapped reads (199,109,142 for A01 vs. 23,024,914 for A04), enrichment efficiency (19.79% for A01 vs. 1.71% for A04), sequencing genome coverage (68.06% for A01 vs. 21.65% for A04), among others (Please see Data S4).

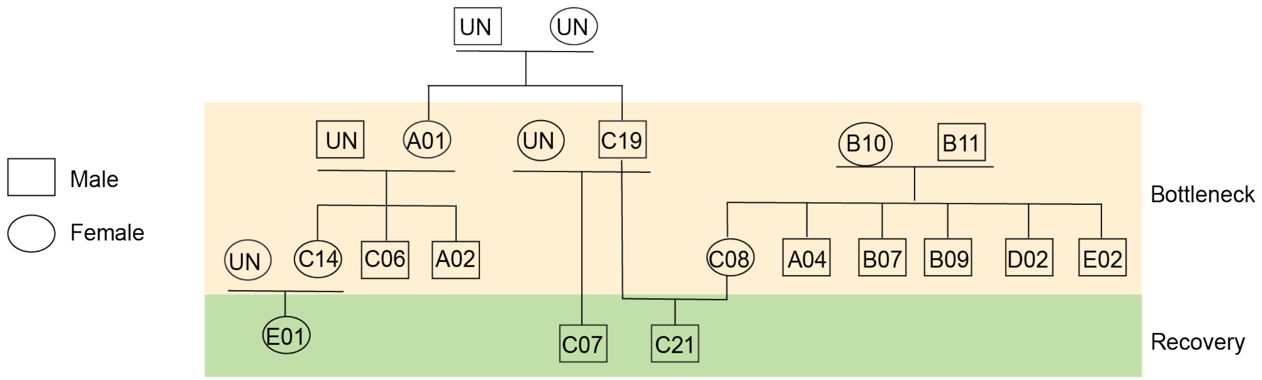

Fig. S11. The pedigree of studied Hainan gibbons constructed using the fecal genomic data. UN denotes an unknown individual: unsampled or uncertain existence in the wild. Male D01 and Female C22 are excluded from this analysis because the two individuals are beyond third-degree relationships with other individuals.

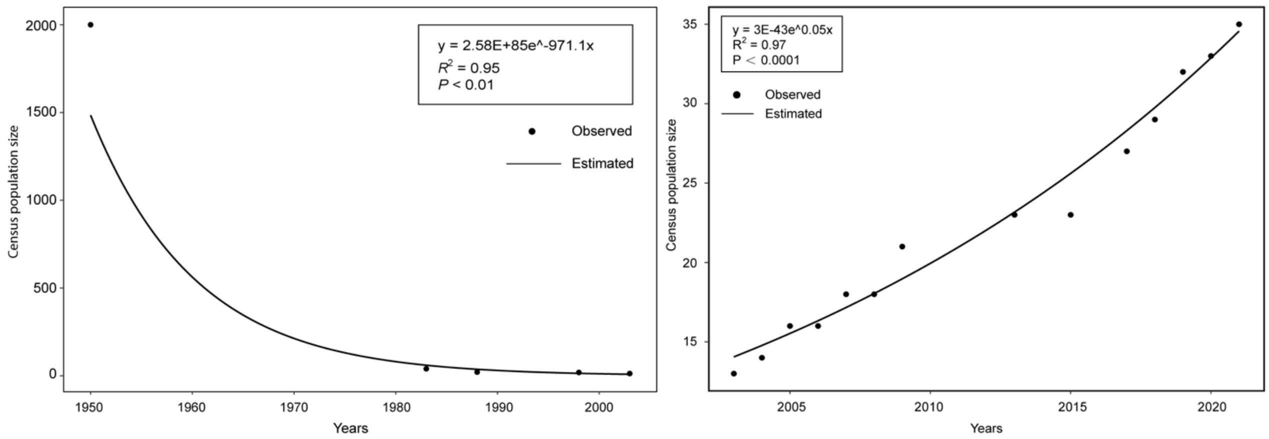

Fig. S12. Changes in Hainan gibbon census population sizes from 1950 to the present (Materials and Methods).

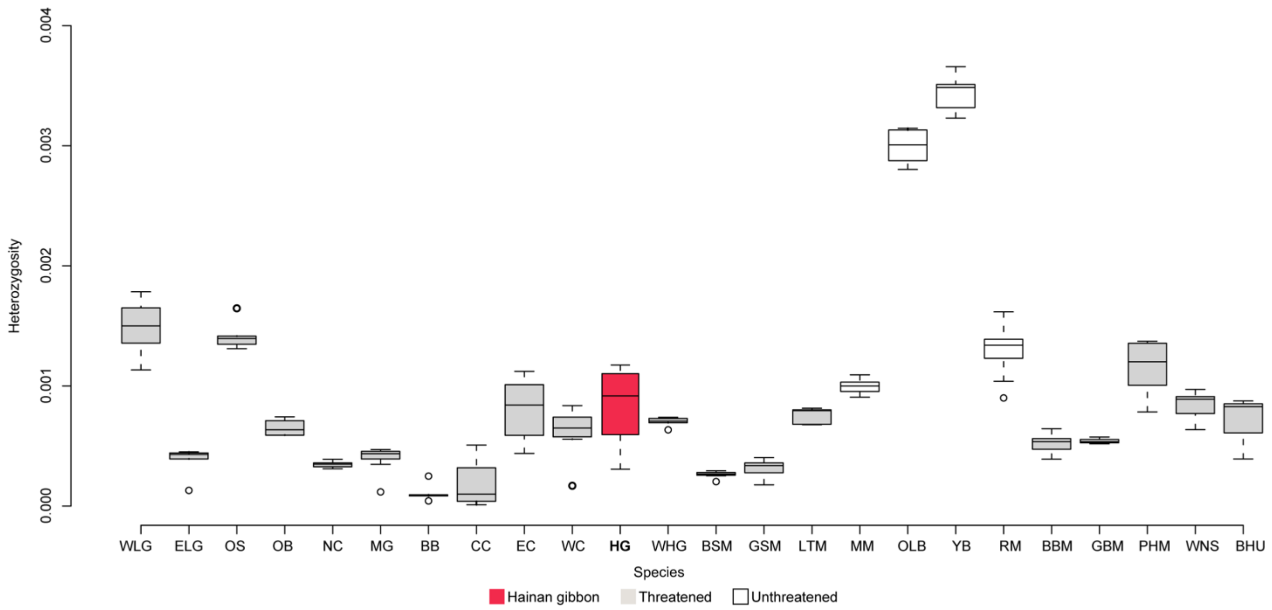

Fig. S13. Genomic genetic diversity of 24 primate species. WLG: Western lowland gorilla, ELG: Eastern lowland gorilla, OS: Sumatran orangutan, OB: Bornean orangutan, NC: Nigeria-Cameroon chimpanzee, MG: Mountain gorilla, BB: Bonobo, CC: Central chimpanzee, EC: Eastern chimpanzee, WC: Western chimpanzee, HG: Hainan gibbon, WHG: Western hoolock gibbon, BSM: Black sub-nosed monkey, GSM: Golden sub-nosed monkey, LTM: Lion-tailed macaque, MM: Mona monkey, OLB: Olive baboon, YB: Yellow baboon, RM: Rhesus macaque, BBM: Black-faced black spider monkey, GBM: Golden-backed squirrel monkey, PHM: Purus red howler monkey, WNS: White-nosed saki, BHU: Bald-headed uakari.

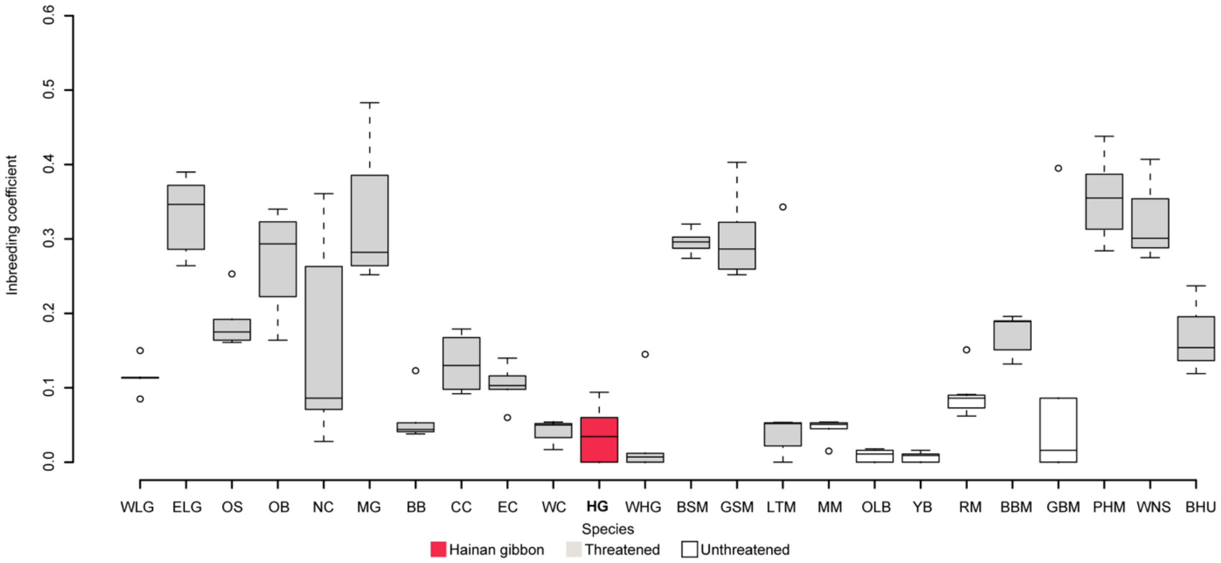

Fig. S14. Inbreeding coefficient estimates of the 24 primate species based on their population genomic data. The abbreviation for each species is the same as in fig. S13.

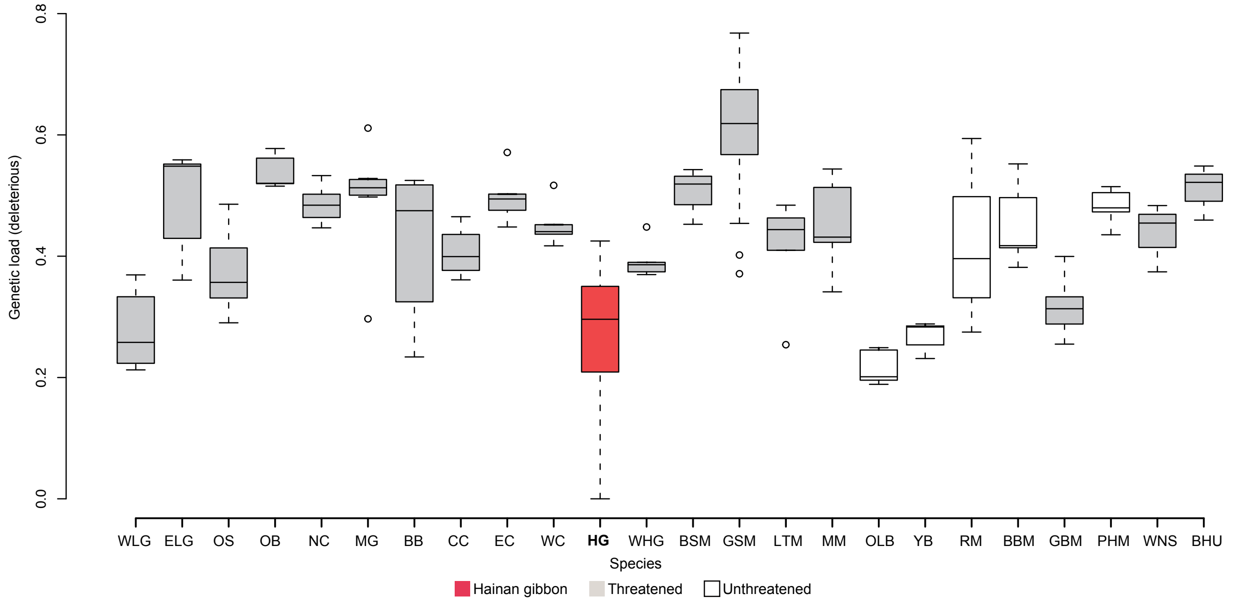

Fig. S15. Genetic load with deleterious mutations in homozygote state for the 24 primate species. The abbreviation for each species is the same as in fig. S13.

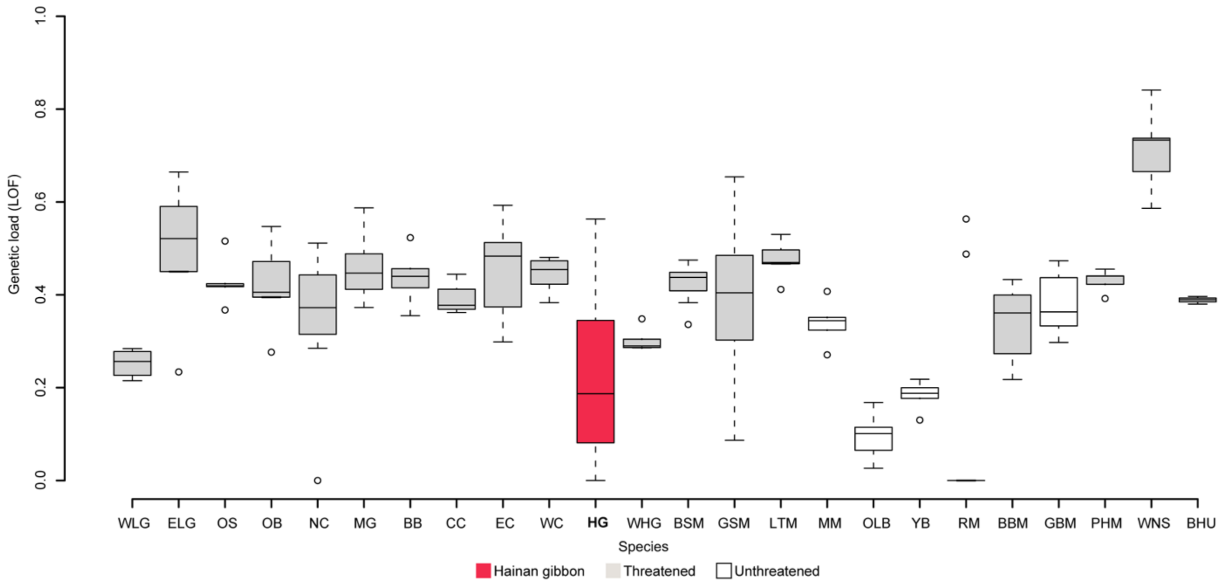

Fig. S16. Genetic load with LoF mutations in homozygote state for the 24 primate species. The abbreviation for each species is the same as in fig. S13.

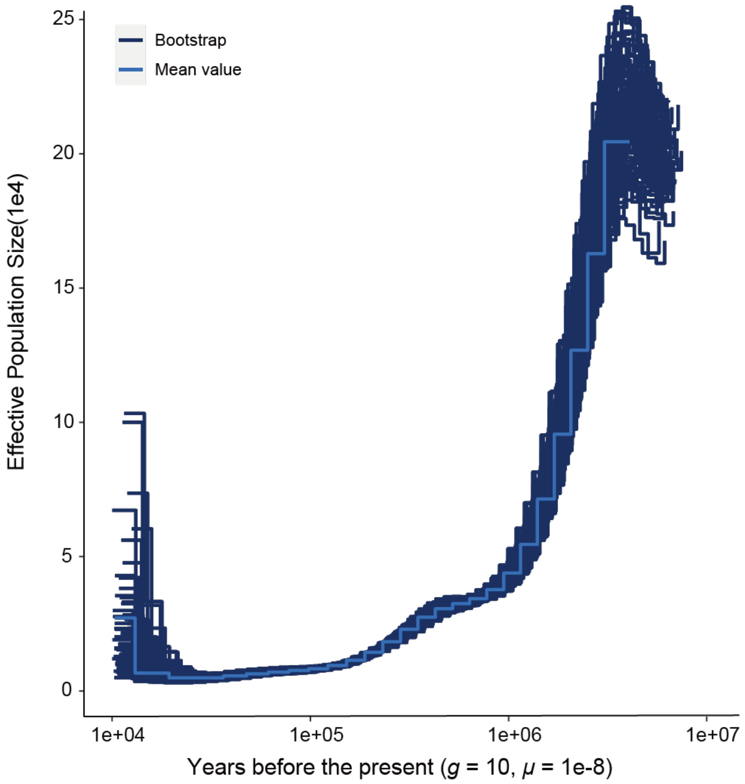

Fig. S17. Reconstruction of the Hainan gibbon demographic history using the PSMC with 200 bootstraps. The shaded region indicates the 95% confidential interval (CI).

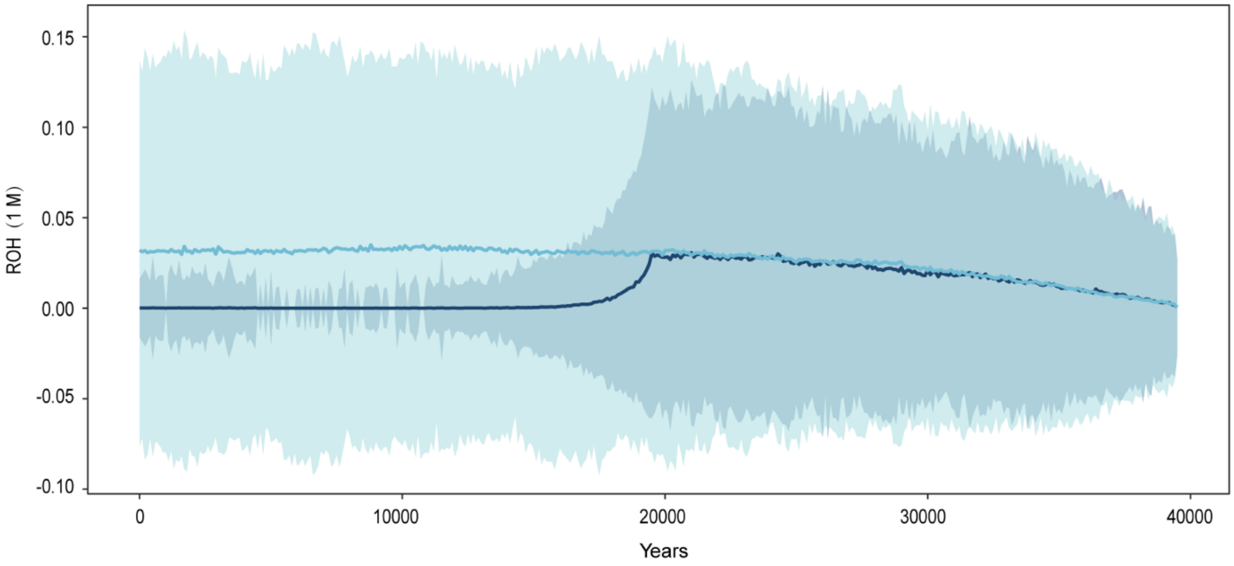

Fig. S18. Accumulation trajectories of inbreeding levels indexed by ROH (1M). Dark blue line: accumulation trajectories based on the Hainan gibbon's demographic history reconstructed in our study; Light blue line: accumulation trajectories based on a simulated demographic history for a classical island small population, which is similar to the reconstructed Hainan gibbon's demographic history but does not have a recent historical population expansion. The solid line and the shaded region denote the mean and 95% confidence interval of the inference, respectively. ROH (runs of homozygosity) symbols inbreeding level.

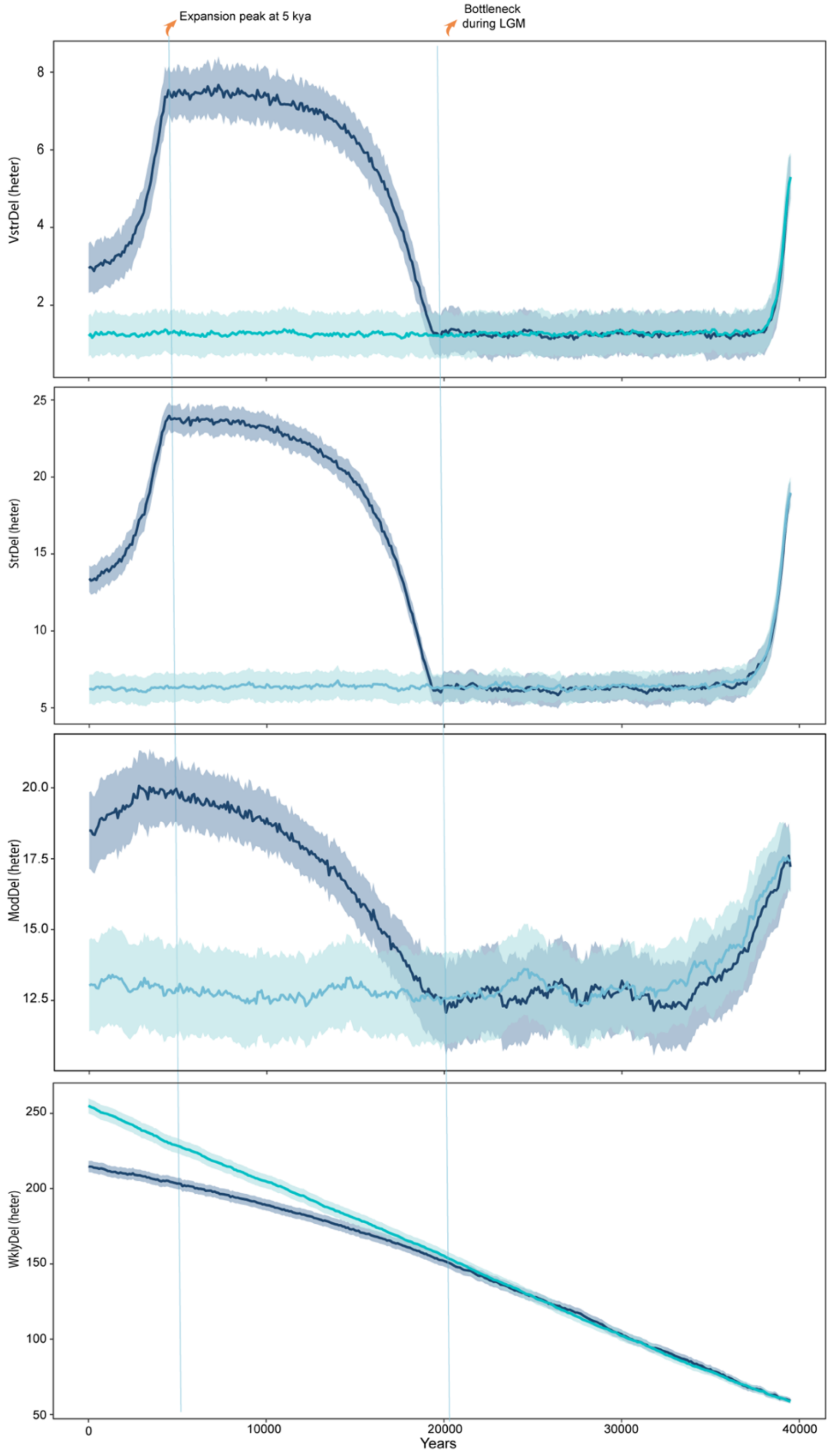

Fig. S19. Simulated accumulation trajectories of genetic load levels in heterozygous state based on the gibbon demographic history inferred in this study or the island small population model without the recent 5 kya expansion. The meanings of dark blue and light blue lines are the same as in fig. S16. The solid line and the shaded regions denote the mean and 95% confidence interval of the inference, respectively. heter means in heterozygous state. VStrDel, StrDel, MoDel and WkDel mean very strong ($s < -0.1$), strongly ($-0.1 < s < -0.01$), moderately ($-0.01 < s < -0.001$) and weakly deleterious mutations ($s \geq -0.001$), respectively.

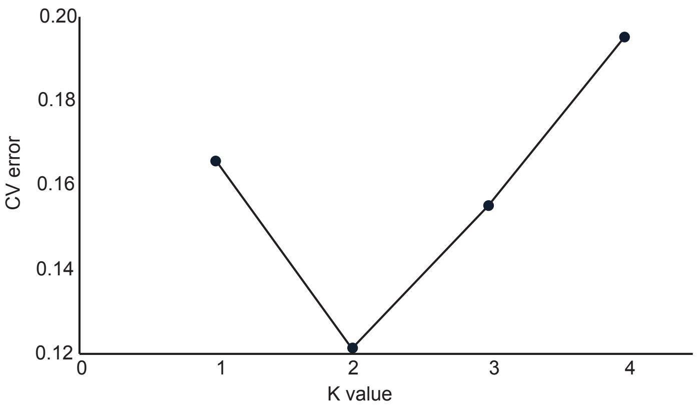

Fig. S20. The rate of change in cross-validation error between successive *K*-values (ranging from 1 to 4).

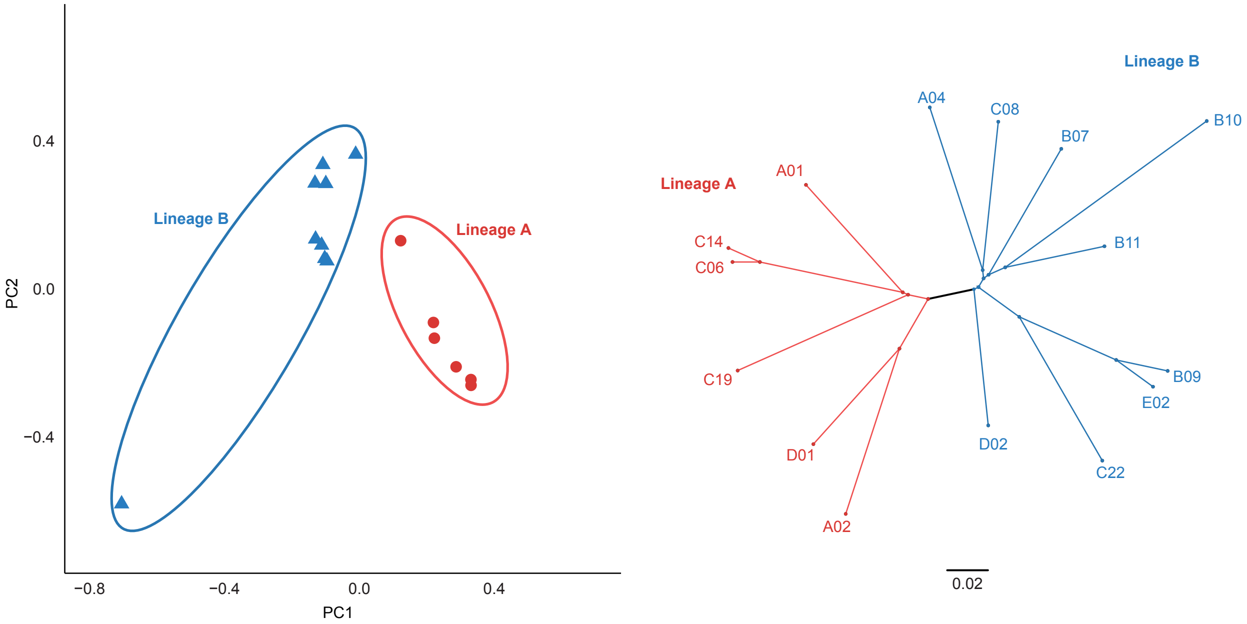

Fig. S21. Principal components analysis and neighbor-joining tree based on pairwise genetic distances, showing genetic differentiation between the two gibbon lineages.

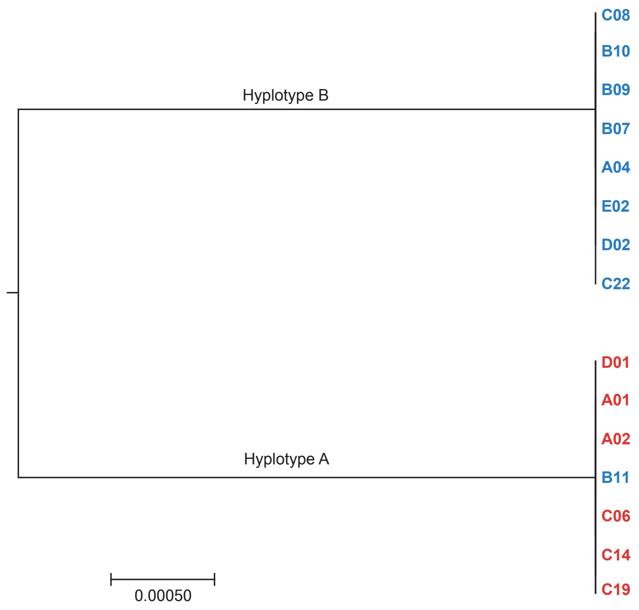

Fig. S22. Maximum likelihood tree reconstructed using the mitochondrial D-loop DNA sequences from gibbon individuals during the decline stage of the Hainan gibbon population. Individuals highlighted in red correspond to Lineage A, and those in blue correspond to Lineage B, as defined by nuclear genome analyses.

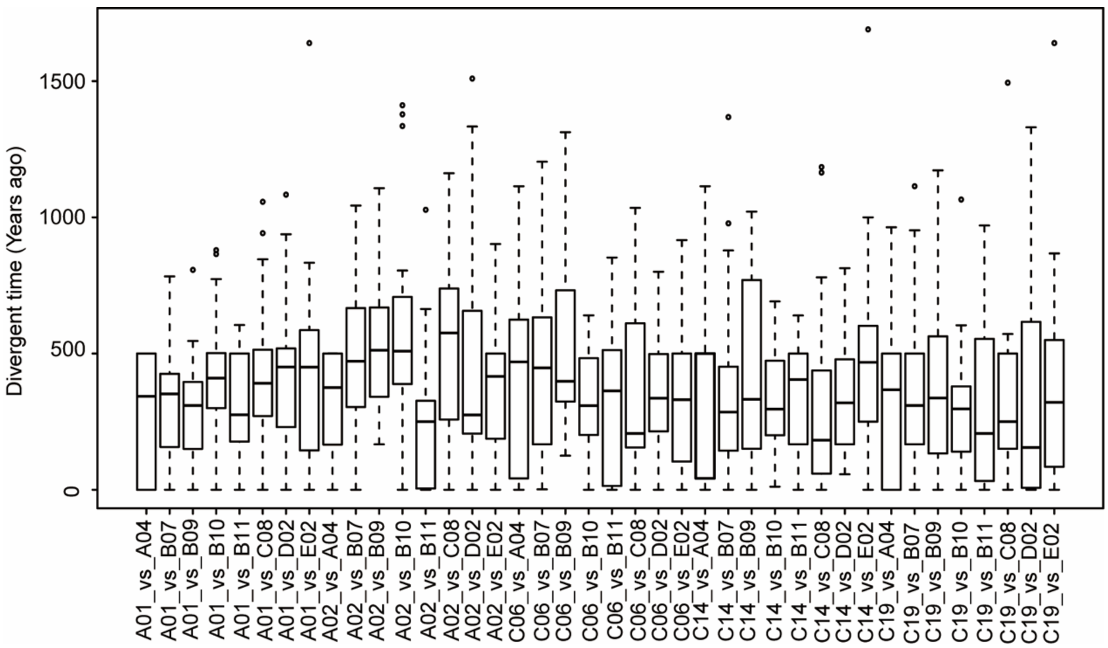

Fig. S23. Divergent time estimation between Lineages A and B of the Hainan gibbon using a Coalescent Hidden Markov model.

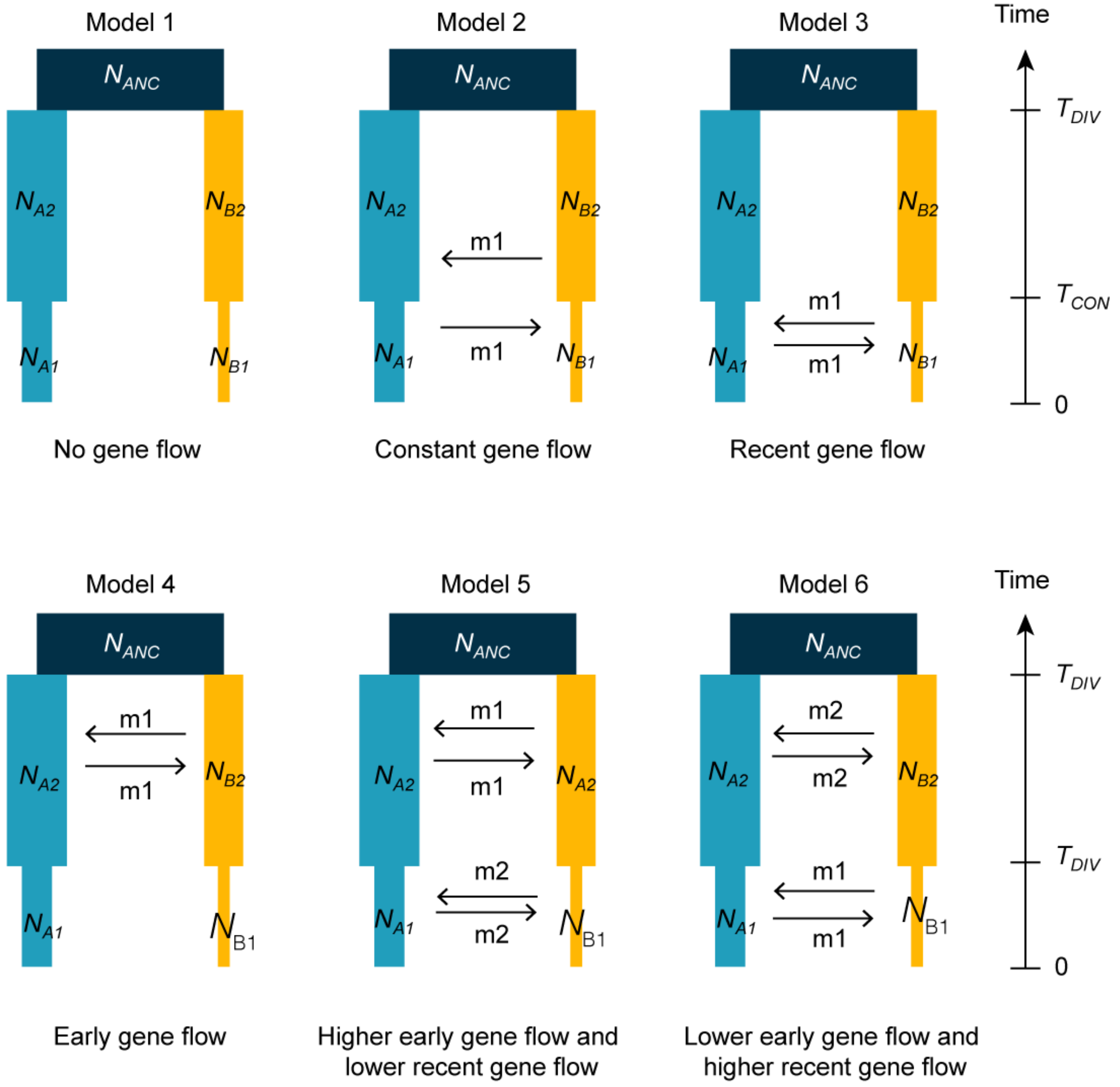

Fig. S24. Six evolutionary scenarios for the divergence time estimation between Lineages A and B using *fastsimcoal2*. The ranges for each parameter are set as: N_{A1} (200 ~ 500), N_{B1} (100 ~ 450), N_{A2} (1000 ~ 2000), N_{B2} (9500 ~ 11000), N_{ANC} (12000 ~ 14000), T_{CON} (90 ~ 200), T_{DIV} (400 ~ 600), *m1* (0.001 ~ 0.01), *m2* (1E-05 ~ 1E-03). "*N*" is the effective population size. "*T*" is the divergence or contraction time. "*m*" is the gene flow rate and "*ANC*" is the ancestral state.

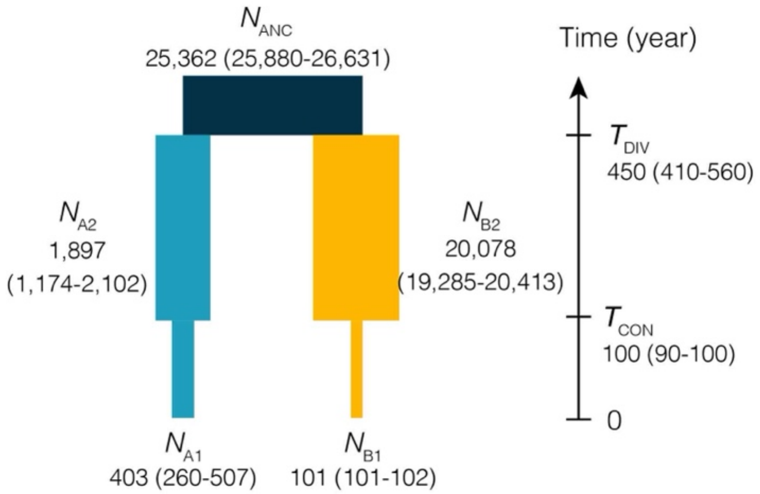

Fig. S25. The best model (Model 1, the highest likelihood) for the divergence time estimation between Lineages A and B in the *fastsimcoal2* simulation (100 times with each of the 100 bootstrapped SFS data). "*T*" is the estimated divergence time; "*N*" is the effective population size. "*ANC*" is the ancestral state.

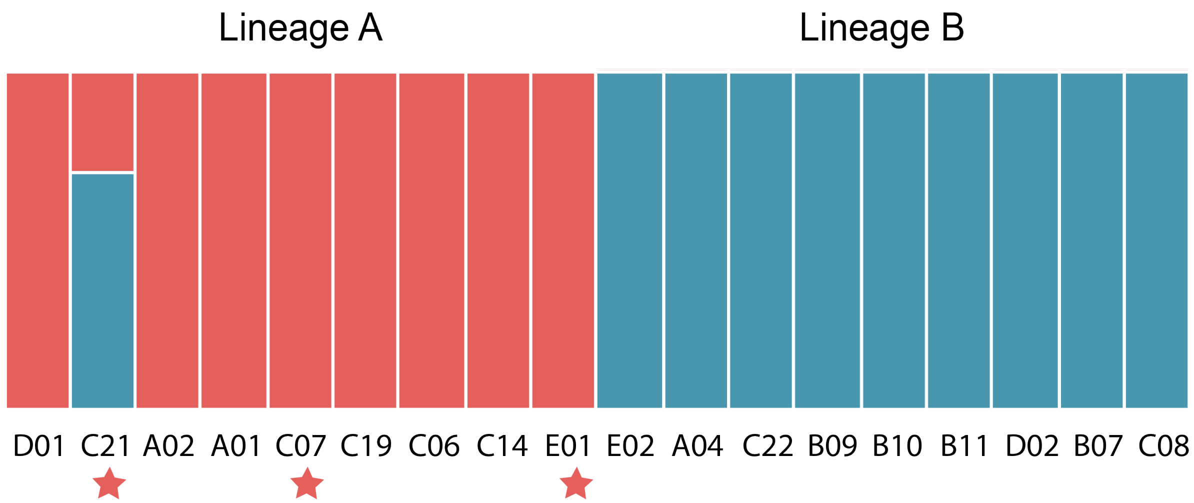

Fig. S26. The population structure of the Hainan gibbon population (*K* = 2). All the 18 Hainan gibbon individuals were shown. *Offspring in Family group C.

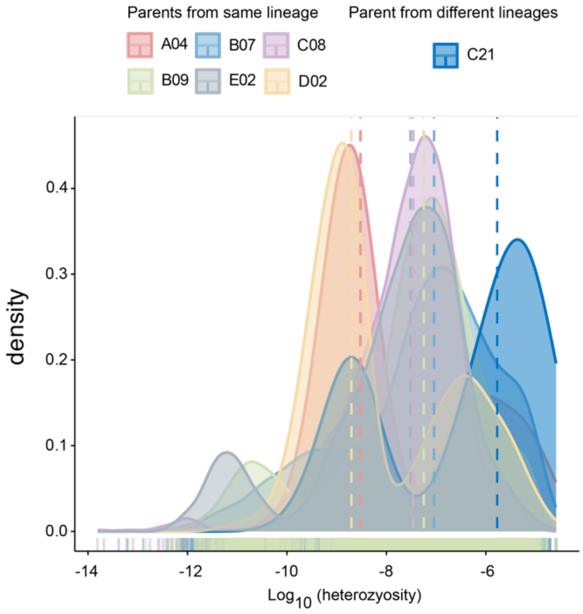

Fig. S27. Heterozygosity distribution difference (1M window size) between hybrid offspring from different lineages and those from the same lineage. Dashed lines denote median.

Table S1. Fecal samples of the Hainan gibbon collected in this study.

Family Group	Sample ID	Location	Collection Date
Group A	A-01	Daankou	18/5/2017
	A-03	Honghegu	27/6/2017
	A-05	Honghegu	27/6/2017
	A-02	Honghegu	27/6/2017
	A-04	Honghegu	27/6/2017
	A-06	Honghegu	27/6/2017
Group B	B-01	Hengganggou	3/6/2017
	B-04	Nanbangou	4/6/2017
	B-08	Nanbangou	5/6/2017
	B-10	Nanbangou	6/6/2017
	B-02	Hengganggou	3/6/2017
	B-03	Nanbangou	4/6/2017
	B-05	Nanbangou	4/6/2017
	B-09	Nanbangou	5/6/2017
	B-06	Nanbangou	5/6/2017
	B-11	Nanbangou	5/6/2017
	B-07	Nanbangou	5/6/2017
Group C	C-01	Miaocun	18/2/2017
	C-07	Miaocun	3/3/2017
	C-15	Miaocun	3/3/2017
	C-02	Miaocun	18/2/2017
	C-19	Miaocun	7/3/2017
	C-03	Miaocun	18/2/2017
	C-06	Miaocun	3/3/2017
	C-17	Miaocun	6/3/2017
	C-05	Miaocun	28/2/2017
	C-10	Miaocun	3/3/2017
	C-14	Miaocun	3/3/2017
	C-13	Miaocun	3/3/2017
	C-08	Miaocun	6/3/2017
	C-09	Miaocun	7/3/2017
	C-11	Miaocun	3/3/2017
	C-12	Miaocun	3/3/2017
	C-16	Miaocun	6/3/2017
	C-18	Miaocun	6/3/2017
	C-20	Miaocun	7/3/2017
	C-21	Miaocun	21/1/2022
	C-22	Miaocun	22/1/2021
Group D	D-01	Kuiyegang	22/9/2020
	D-02	Kuiyegang	10/10/2020
Group E	E-01	Dongbengling	13/9/2020

Table S2. Individual ID and sex for each individual identified in this study.

Fecal sample ID	Individual ID	Sex
A-01, A-03, A-05	A01	Female
A-04, A-06	A04	Male
A-02	A02	Male
B-10, B-01, B-04, B-08	B10	Female
B-07	B07	Male
B-09, B-05, B-03, B-02	B09	Male
B-11, B-06	B11	Male
C-08, C-09, C-11, C-12, C-16, C-18, C-20	C08	Female
C-14, C-05, C-10, C-13	C14	Female
C-19, C-02	C19	Male
C-07, C-01, C-15	C07	Male
C-06, C-03, C-17	C06	Male
C-21	C21	Male
C-22	C22	Female
E-01	E01	Female
E-02	E02	Male
D-01	D01	Female
D-02	D02	Male

Table S3. The equations of Solow growth model and raw sequencing data amount for each Hainan gibbon individual.

Sample ID	Equation	Raw sequencing data amount (Gb)
A01	$y = 1.43 - e^{(-0.028x+0.360)}$	150.93
A02	$y = 0.73 - e^{(-0.0125x-0.323)}$	200.33
A04	$y = 0.43 - e^{(-0.0129x-0.846)}$	165.88
B07	$y = 2.02 - e^{(-0.0224x-0.698)}$	101.32
B09	$y = 1.51 - e^{(-0.016x-0.405)}$	103.09
B10	$y = 0.87 - e^{(-0.028x-0.145)}$	92.34
B11	$y = 0.65 - e^{(-0.021x-0.457)}$	126.83
C06	$y = 0.67 - e^{(-0.023x-0.418)}$	167.67
C07	$y = 0.89 - e^{(-0.0144x-0.130)}$	98.61
C08	$y = 1.03 - e^{(-0.021x-0.003)}$	103.57
C14	$y = 0.86 - e^{(-0.0222x-0.214)}$	101.91
C19	$y = 0.91 - e^{(-0.008x-0.126)}$	212.51
D01	$y = 0.43 - e^{(-0.027x-0.865)}$	331.19
D02	$y = 0.65 - e^{(-0.032x-0.435)}$	284.22
E01	$y = 1.18 - e^{(-0.074x+0.070)}$	250.29
E02	$y = 0.42 - e^{(-0.033x-0.876)}$	144.40
C21	$y = 0.63 - e^{(-0.026x-0.474)}$	202.12
C22	$y = 0.21 - e^{(-0.030x-1.548)}$	201.89

Table S4. Genome-wide diversity comparisons between the Hainan gibbon and other primates.

Species 1	Species 2	Threatened status	P value
Hainan gibbon	Western lowland gorilla		0.0009***
Hainan gibbon	Eastern lowland gorilla		0.0092**
Hainan gibbon	Bornean orangutan		0.2958
Hainan gibbon	Sumatran orangutan		0.0002***
Hainan gibbon	Eastern chimpanzee		0.8490
Hainan gibbon	Nigeria-Cameroon chimpanzee		0.0011**
Hainan gibbon	Western chimpanzee		0.1172
Hainan gibbon	Bonobos		0.0002***
Hainan gibbon	Central chimpanzee		0.0058**
Hainan gibbon	Mountain gorilla	Threatened	0.0046**
Hainan gibbon	Western Hoolock Gibbon		0.2460
Hainan gibbon	Golden sub-nosed monkey		<0.0001****
Hainan gibbon	Black sub-nosed monkey		0.0004***
Hainan gibbon	Lion-tailed Macaque		0.2460
Hainan gibbon	Bald-headed Uakari		0.09454
Hainan gibbon	Purús Red Howler		0.3643
Hainan gibbon	White-nosed Saki		0.0592
Hainan gibbon	Black-faced Black Spider Monkey		0.3643
Hainan gibbon	Mona monkey		0.0002***
Hainan gibbon	Yellow baboon		0.0002***
Hainan gibbon	Olive baboon	Unthreatened	0.0002***
Hainan gibbon	Rhesus macaque		0.0003***
Hainan gibbon	Golden-backed Squirrel Monkey		0.1433

Note: Statistical significance assessed using a *Wilcoxon Rank Sum* Test. *$p \leq 0.05$, ** $p \leq 0.01$, *** $p \leq 0.001$, ****$p \leq 0.0001$, ns: no significant difference.

Table S5. Genome-wide inbreeding level comparisons between the Hainan gibbon and other primates.

Species 1	Species 2	Threatened status	*P* value
Hainan gibbon	Western lowland gorilla		0.0038^{**}
Hainan gibbon	Eastern lowland gorilla		0.0013^{**}
Hainan gibbon	Bornean orangutan		0.0056^{**}
Hainan gibbon	Sumatran orangutan		0.0026^{**}
Hainan gibbon	Eastern chimpanzee		0.0067^{**}
Hainan gibbon	Nigeria-Cameroon chimpanzee		0.0050^{**}
Hainan gibbon	Western chimpanzee		0.5802
Hainan gibbon	Bonobos		0.3250
Hainan gibbon	Central chimpanzee		0.0086^{**}
Hainan gibbon	Mountain gorilla	Threatened	0.0007^{***}
Hainan gibbon	Western Hoolock Gibbon		0.6625
Hainan gibbon	Golden sub-nosed monkey		0.0004^{***}
Hainan gibbon	Black sub-nosed monkey		0.0004^{***}
Hainan gibbon	Lion-tailed Macaque		0.6211
Hainan gibbon	Bald-headed Uakari		0.0026^{**}
Hainan gibbon	Purús Red Howler		0.0137^{*}
Hainan gibbon	White-nosed Saki		0.0026^{**}
Hainan gibbon	Black-faced Black Spider Monkey		0.0172^{*}
Hainan gibbon	Mona monkey		0.4990
Hainan gibbon	Yellow baboon		0.2316
Hainan gibbon	Olive baboon	Unthreatened	0.2361
Hainan gibbon	Rhesus macaque		0.0003^{***}
Hainan gibbon	Golden-backed Squirrel Monkey		0.9500

Note: Statistical significance assessed using a *Wilcoxon Rank Sum* Test. $*p \leq 0.05$, $** p \leq 0.01$, $*** p \leq 0.001$, $****p \leq 0.0001$, ns: no significant difference.

Table S6. Genetic load (LoF) comparisons between Hainan gibbon and other primates.

Species 1	Species 2	Threatened status	*P* value
Hainan gibbon	Western lowland gorilla		0.3434
Hainan gibbon	Eastern lowland gorilla		0.0046**
Hainan gibbon	Bornean orangutan		0.0101*
Hainan gibbon	Sumatran orangutan		0.0050**
Hainan gibbon	Eastern chimpanzee		0.0176*
Hainan gibbon	Nigeria-Cameroon chimpanzee		0.0316*
Hainan gibbon	Western chimpanzee		0.0025**
Hainan gibbon	Bonobos		0.0050**
Hainan gibbon	Central chimpanzee		0.0425*
Hainan gibbon	Mountain gorilla	Threatened	0.0023**
Hainan gibbon	Western Hoolock Gibbon		0.2020
Hainan gibbon	Golden sub-nosed monkey		0.0022
Hainan gibbon	Black sub-nosed monkey		0.0031**
Hainan gibbon	Lion-tailed Macaque		0.0058*
Hainan gibbon	Bald-headed Uakari		0.0424**
Hainan gibbon	Purús Red Howler		0.0057**
Hainan gibbon	White-nosed Saki		0.0057
Hainan gibbon	Black-faced Black Spider Monkey		0.0740
Hainan gibbon	Mona monkey		0.05132
Hainan gibbon	Yellow baboon		0.8710
Hainan gibbon	Olive baboon	Unthreatened	0.4168
Hainan gibbon	Rhesus macaque		0.1269
Hainan gibbon	Golden-backed Squirrel Monkey		0.03476*

Note: Statistical significance assessed using a *Wilcoxon Rank Sum* Test. *$p \le 0.05$, ** $p \le 0.01$, *** $p \le 0.001$, ****$p \le 0.0001$, ns: no significant difference.

Table S7. Genetic load (deleterious mutations) comparisons between the Hainan gibbon and other primates.

Species 1	Species 2	Threatened status	*P* value
Hainan gibbon	Western lowland gorilla		1.0000
Hainan gibbon	Eastern lowland gorilla		0.0009***
Hainan gibbon	Bornean orangutan		0.0006***
Hainan gibbon	Sumatran orangutan		0.0992
Hainan gibbon	Eastern chimpanzee		0.0006***
Hainan gibbon	Nigeria-Cameroon chimpanzee		<0.0001****
Hainan gibbon	Western chimpanzee		0.0013**
Hainan gibbon	Bonobos		0.0992
Hainan gibbon	Central chimpanzee		0.0359*
Hainan gibbon	Mountain gorilla	Threatened	0.0019**
Hainan gibbon	Western Hoolock Gibbon		0.0399*
Hainan gibbon	Golden sub-nosed monkey		<0.0001****
Hainan gibbon	Black sub-nosed monkey		0.0004***
Hainan gibbon	Lion-tailed Macaque		0.0320*
Hainan gibbon	Bald-headed Uakari		0.0142*
Hainan gibbon	Purús Red Howler		0.0027**
Hainan gibbon	White-nosed Saki		0.0346*
Hainan gibbon	Black-faced Black Spider Monkey		0.0120**
Hainan gibbon	Mona monkey		0.0120**
Hainan gibbon	Yellow baboon		0.7595
Hainan gibbon	Olive baboon	Unthreatened	0.0982
Hainan gibbon	Rhesus macaque		0.0306*
Hainan gibbon	Golden-backed Squirrel Monkey		0.5815

Note: Statistical significance assessed using a *Wilcoxon Rank Sum* Test. *$p \leq 0.05$, ** $p \leq 0.01$, *** $p \leq 0.001$, ****$p \leq 0.0001$, ns: no significant difference.

Table S8. Simulation parameters used for Model 1 in *fastsimcoal2* with bootstrapped site frequency spectrum data.

Variable	Parameters	Minimum	Maximum
Effective population size	Recent Lineage A (N_{A1})	260	507
	Recent Lineage B (N_{B1})	101	102
	Early Lineage A (N_{A2})	1,174	2,102
	Early Lineage B (N_{B2})	19,285	20,413
	Ancestor of Lineages A and B (N_{ANC})	25,880	26,631
Generation time of historical events	Contraction time of Lineages A and B (T_{CON}/10 year per generation)	9	10
	Divergence between Lineages A and B (T_{DIV}/10 year per generation)	41	66

Data S1. Museum and blood samples used in this study.

Data S2. Microsatellite genotype of identified Hainan gibbon individuals.

Data S3. Enrichment fold of host DNA enriched from fecal DNA extracts.

Data S4. Effectiveness of host DNA enrichment and genome sequencing statistics.

Data S5. Genome sequencing summary statistics of the museum samples.

Data S6. NCBI accession number, reference genomes, and SNP filtering criteria of 23 primate species used in this study.

Data S7. Summary of population genetic estimates for the 24 focal primate species.

Data S8. Functional annotations of balancing selected genes in the Hainan gibbon population.

Data S9. Variable sites on the 880 bp mitochondrial D-loop regions in the studied Hainan gibbons.

Data S10. Genome-wide heterozygosity for each of the 13 Hainan gibbon individuals at different sequencing depths.

Data S11. Inbreeding coefficient for each of the 13 Hainan gibbon individuals at different sequencing depths.

Data S12. Genetic load estimates in deleterious mutations and LoF for each of the 13 Hainan gibbon individuals at different sequencing depths.

REFERENCES

1. J. Tollefson, Humans are driving one million species to extinction. *Nature* **569**, 171 (2019).

2. International Union for Conservation of Nature (IUCN), "Table 7: Species changing IUCN Red List Status" (IUCN, 2025); www.iucnredlist.org/resources/summary-statistics#Table%20 7.

3. M. Hoffmann, C. Hilton-Taylor, A. Angulo, M. Böhm, T. M. Brooks, S. H. M. Butchart, K. E. Carpenter, J. Chanson, B. Collen, N. A. Cox, W. R. T. Darwall, N. K. Dulvy, L. R. Harrison, V. Katariya, C. M. Pollock, S. Quader, N. I. Richman, A. S. L. Rodrigues, M. F. Tognelli, J.-C. Vié, J. M. Aguiar, D. J. Allen, G. R. Allen, G. Amori, N. B. Ananjeva, F. Andreone, P. Andrew, A. L. A. Ortiz, J. E. M. Baillie, R. Baldi, B. D. Bell, S. D. Biju, J. P. Bird, P. Black-Decima, J. J. Blanc, F. Bolaños, W. Bolivar-G, I. J. Burfield, J. A. Burton, D. R. Capper, F. Castro, G. Catullo, R. D. Cavanagh, A. Channing, N. L. Chao, A. M. Chenery, F. Chiozza, V. Clausnitzer, N. J. Collar, L. C. Collett, B. B. Collette, C. F. C. Fernandez, M. T. Craig, M. J. Crosby, N. Cumberlidge, A. Cuttelod, A. E. Derocher, A. C. Diesmos, J. S. Donaldson, J. W. Duckworth, G. Dutson, S. K. Dutta, R. H. Emslie, A. Farjon, S. Fowler, J. Freyhof, D. L. Garshelis, J. Gerlach, D. J. Gower, T. D. Grant, G. A. Hammerson, R. B. Harris, L. R. Heaney, S. B. Hedges, J.-M. Hero, B. Hughes, S. A. Hussain, M. Javier Icochea, R. F. Inger, N. Ishii, D. T. Iskandar, R. K. B. Jenkins, Y. Kaneko, M. Kottelat, K. M. Kovacs, S. L. Kuzmin, E. L. Marca, J. F. Lamoreux, M. W. N. Lau, E. O. Lavilla, K. Leus, R. L. Lewison, G. Lichtenstein, S. R. Livingstone, V. Lukoschek, D. P. Mallon, P. J. K. McGowan, A. M. Ivor, P. D. Moehlman, S. Molur, A. M. Alonso, J. A. Musick, K. Nowell, R. A. Nussbaum, W. Olech, N. L. Orlov, T. J. Papenfuss, G. Parra-Olea, W. F. Perrin, B. A. Polidoro, M. Pourkazemi, P. A. Racey, J. S. Ragle, M. Ram, G. Rathbun, R. P. Reynolds, A. G. J. Rhodin, S. J. Richards, L. O. Rodríguez, S. R. Ron, C. Rondinini, A. B. Rylands, Y. S. de Mitcheson, J. C. Sanciangco, K. L. Sanders, G. Santos-Barrera, J. Schipper, C. Self-Sullivan, Y. Shi, A. Shoemaker, F. T. Short, C. Sillero-Zubiri, D. L. Silvano, K. G. Smith, A. T. Smith, J. Snoeks, A. J. Stattersfield, A. J. Symes, A. B. Taber, B. K. Talukdar, H. J. Temple, R. Timmins, J. A. Tobias, K. Tsytsulina, D. Tweddle, C. Ubeda, S. V. Valenti, P. P. van Dijk, L. M. Veiga, A. Veloso, D. C. Wege, M. Wilkinson, E. A. Williamson, F. Xie, B. E. Young, H. R. Akçakaya, L. Bennun, T. M. Blackburn, L. Boitani, H. T. Dublin, G. A. B. da Fonseca, C. Gascon, T. E. Lacher Jr., G. M. Mace, S. A. Mainka, J. A. McNeely, R. A. Mittermeier, G. McGregor Reid, J. P. Rodriguez, A. A. Rosenberg, M. J.

Samways, J. Smart, B. A. Stein, S. N. Stuart, The impact of conservation on the status of the world's vertebrates. *Science* **330**, 1503–1509 (2010).

4. J. von Seth, N. Dussex, D. Díez-Del-Molino, T. van der Valk, V. E. Kutschera, M. Kierczak, C. C. Steiner, S. Liu, M. T. P. Gilbert, M.-H. S. Sinding, S. Prost, K. Guschanski, S. K. S. S. Nathan, S. Brace, Y. L. Chan, C. W. Wheat, P. Skoglund, O. A. Ryder, B. Goossens, A. Götherström, L. Dalén, Genomic insights into the conservation status of the world's last remaining *Sumatran rhinoceros* populations. *Nat. Commun.* **12**, 2393 (2021).

5. T. V. D. Valk, D. Diez-Del-Molino, T. Marques-Bonet, K. Guschanski, L. Dalen, Historical genomes reveal the genomic consequences of recent population decline in eastern gorillas. *Curr. Biol.* **29**, 165–170.e6 (2019).

6. R. Frankham, Conservation genetics. *Annu. Rev. Genet.* **29**, 305–327 (1995).

7. K. M. Cammen, T. F. Schultz, W. D. Bowen, M. O. Hammill, W. B. Puryear, J. Runstadler, F. W. Wenzel, S. A. Wood, M. Kinnison, Genomic signatures of population bottleneck and recovery in Northwest Atlantic pinnipeds. *Ecol. Evol.* **8**, 6599–6614 (2018).

8. Y. B. Simons, M. C. Turchin, J. K. Pritchard, G. Sella, The deleterious mutation load is insensitive to recent population history. *Nat. Genet.* **46**, 220–224 (2014).

9. F. C. Ceballos, K. Gürün, N. E. Altınışık, H. C. Gemici, C. Karamurat, D. Koptekin, K. B. Vural, I. Mapelli, E. Sağlıcan, E. Sürer, Y. S. Erdal, A. Götherström, F. Özer, Ç. Atakuman, M. Somel, Human inbreeding has decreased in time through the Holocene. *Curr. Biol.* **31**, 3925–3934 (2021).

10. A. R. Whiteley, S. W. Fitzpatrick, W. C. Funk, D. A. Tallmon, Genetic rescue to the rescue. *Trends Ecol. Evol.* **30**, 42–49 (2015).

11. W. Y. Chan, A. A. Hoffmann, M. J. H. Oppen, Hybridization as a conservation management tool. *Conserv. Lett.* **12**, e12652 (2019).

12. D. A. Tallmon, G. Luikart, R. S. Waples, The alluring simplicity and complex reality of genetic rescue. *Trends Ecol. Evol.* **19**, 489–496 (2004).

13. R. Frankham, J. D. Ballou, M. D. B. Eldridge, R. C. Lacy, K. Ralls, M. R. Dudash, C. B. Fenster, Predicting the probability of outbreeding depression. *Conserv. Biol.* **25**, 465–475 (2011).

14. P. W. Hedrick, J. A. Robinson, R. O. Peterson, J. A. Vucetich, Genetics and extinction and the example of Isle Royale wolves. *Anim. Conserv.* **22**, 302–309 (2019).

15. J. Zhou, F. Wei, M. Li, J. Zhang, D. Wang, R. Pan, Hainan black-crested gibbon is headed for extinction. *Int. J. Primatol.* **26**, 453–465 (2005).

16. G. Liu, X. Liu, Z. Liu, X. Zhi, X. Qi, J. Zhou, X. Hong, Y. Mo, B. P. L. Chan, C. A. Chapman, Z. Jiang, The critically endangered Hainan gibbon (*Nomascus hainanus*) population increases but not at the maximum possible rate. *Int. J. Primatol.* **43**, 932–945 (2022).

17. M. A. Zemanova, Noninvasive genetic assessment is an effective wildlife research tool when compared with other approaches. *Genes* **12**, 1672 (2021).

18. Y. Guo, J. Chang, L. Han, T. Liu, G. Li, P. A. Garber, N. Xiao, J. Zhou, The genetic status of the critically endangered Hainan Gibbon (*Nomascus hainanus*): A species moving toward extinction. *Front. Genet.* **11**, 608633 (2020).

19. K. L. Chiou, C. M. Bergey, Methylation-based enrichment facilitates low-cost, noninvasive genomic scale sequencing of populations from feces. *Sci. Rep.* **8**, 1975 (2018).

20. A. Leutenegger, B. Prum, E. Genin, C. Verny, A. Lemainque, F. Clerget-Darpoux, E. A. Thompson, Estimation of the inbreeding coefficient through use of genomic data. *Am. J. Hum. Genet.* **73**, 516–523 (2003).

21. D. C. Presgraves, Recombination enhances protein adaptation in *Drosophila melanogaster*. *Curr. Biol.* **15**, 1651–1656 (2005).

22. M. Rousselle, A. Laverre, E. Figuet, B. Nabholz, N. Galteir, Influence of recombination and GC-biased gene conversion on the adaptive and nonadaptive substitution rate in mammals versus birds. *Mol. Biol. Evol.* **36**, 458–471 (2019).

23. K. M. Siewert, B. F. Voight, Detecting long-term balancing selection using allele frequency correlation. *Mol. Biol. Evol.* **34**, 2996–3005 (2017).

24. A. Roy, Y.-N. Lin, J. E. Agno, F. J. DeMayo, M. M. Matzuk, Tektin 3 is required for progressive sperm motility in mice. *Mol. Reprod. Dev.* **76**, 453–459 (2009).

25. P. Yang, W. Tang, H. Li, R. Hua, Y. Yuan, Y. Zhang, Y. Zhu, Y. Cui, J. Sha, T-complex protein 1 subunit zeta-2 (CCT6B) deficiency induces murine teratospermia. *PeerJ* **9**, e11545 (2021).

26. A. Eudes, G. Mouille, J. Thevenin, A. Goyallon, Z. Minic, L. Jouanin, Purification, cloning and functional characterization of an endogenous beta-glucuronidase in *Arabidopsis thaliana*. *Plant Cell Physiol.* **49**, 1331–1341 (2008).

27. H. Deng, J. Zhou, Thirteen years observation on diet composition of Hainan gibbons (*Nomascus hainanus*). *North west. J. Zool.* **14**, e171703 (2018).

28. T. Mailund, J. Y. Dutheil, A. Hobolth, G. Lunter, M. H. Schierup, Estimating divergence time and ancestral effective population size of Bornean and Sumatran orangutan subspecies using a coalescent hidden Markov model. *PLOS Genet.* **7**, e1001319 (2011).

29. J. A. Robinson, C. C. Kyriazis, S. F. Nigenda-Morales, A. C. Beichman, L. Rojas-Bracho, K. M. Robertson, M. C. Fontaine, R. K. Wayne, K. E. Lohmueller, B. L. Taylor, P. A. Morin, The critically endangered vaquita is not doomed to extinction by inbreeding depression. *Science* **376**, 635–639 (2022).

30. N. Snyder-Mackler, W. H. Majoros, M. L. Yuan, A. O. Shaver, J. B. Gordon, G. H. Kopp, S. A. Schlebusch, J. D. Wall, S. C. Alberts, S. Mukherjee, X. Zhou, J. Tung, Efficient genome-wide sequencing and low-coverage pedigree analysis from noninvasively collected samples. *Genetics 203*, 699–714 (2016).

31. P. Taberlet, L. P. Waits, G. Luikart, Noninvasive genetic sampling: Look before you leap. *Trends Ecol. Evol.* **14**, 323–327 (1999).

32. B. J. Cardinale, J. E. Duffy, A. Gonzalez, D. U. Hooper, C. Perrings, P. Venail, A. Narwani, G. M. Mace, D. Tilman, D. A. Wardle, A. P. Kinzig, G. C. Daily, M. Loreau, J. B. Grace, A. Larigauderie, D. S. Srivastava, S. Naeem, Biodiversity loss and its impact on humanity. *Nature* **486**, 59–67 (2012).

33. J. Louys, P. Roberts, Environmental drivers of megafauna and hominin extinction in Southeast Asia. *Nature* **586**, 402–406 (2020).

34. L. Carbone, R. A. Harris, S. Gnerre, K. R. Veeramah, B. Lorente-Galdos, J. Huddleston, T. J. Meyer, J. Herrero, C. Roos, B. Aken, F. Anaclerio, N. Archidiacono, C. Baker, D. Barrell, M. A. Batzer, K. Beal, A. Blancher, C. L. Bohrson, M. Brameier, M. S. Campbell, O. Capozzi, C. Casola, G. Chiatante, A. Cree, A. Damert, P. J. de Jong, L. Dumas, M. Fernandez-Callejo, P. Flicek, N. V. Fuchs, I. Gut, M. Gut, M. W. Hahn, J. Hernandez-Rodriguez, L. D. W. Hillier, R. Hubley, B. Ianc, Z. Izsvák, N. G. Jablonski, L. M. Johnstone, A. Karimpour-Fard, M. K. Konkel, D. Kostka, N. H. Lazar, S. L. Lee, L. R. Lewis, Y. Liu, D. P. Locke, S. Mallick, F. L. Mendez, M. Muffato, L. V. Nazareth, K. A. Nevonen, M. O'Bleness, C. Ochis, D. T. Odom, K. S. Pollard, J. Quilez, D. Reich, M. Rocchi, G. G. Schumann, S. Searle, J. M. Sikela, G. Skollar, A. Smit, K. Sonmez, B. ten Hallers, E. Terhune, G. W. C. Thomas, B. Ullmer, M. Ventura, J. A. Walker, J. D. Wall, L. Walter, M. C. Ward, S. J. Wheelan, C. W. Whelan, S. White, L. J. Wilhelm, A. E. Woerner, M. Yandell, B. Zhu, M. F. Hammer, T. Marques-Bonet, E. E. Eichler, L. Fulton, C. Fronick, D. M. Muzny, W. C. Warren, K. C. Worley, J. Rogers, R. K. Wilson, R. A. Gibbs, Gibbon genome and the fast karyotype evolution of small apes. *Nature* **513**, 195–201 (2014).

35. J. Chang, D. Chen, W. Liang, Z. Zhang, Molecular demographic history of the Hainan Peacock Pheasant (*Polyplectron katsumatae*) and its conservation implications. *Chin. Sci. Bull.* **58**, 2185–2190 (2013).

36. O. Mazet, W. Rodríguez, S. Grusea, S. Boitard, L. Chikhi, On the importance of being structured: Instantaneous coalescence rates and human evolution—Lessons for ancestral population size inference. *Heredity* **116**, 362–371 (2016).

37. "IUCN Red List Categories and Criteria: Version 3.1," (IUCN Species Survival Commission, IUCN, Gland, Switzerland and Cambridge, UK, 2001).

38. K. Näsvall, J. Boman, L. Höök, R. Vila, C. Wiklund, N. Backström, Nascent evolution of recombination rate differences as a consequence of chromosomal rearrangements. *PLOS Genet.* **19**, e1010717 (2023).

39. K. B. Strier, What does variation in primate behavior mean? *Am. J. Phys. Anthropol.* **162**, 4–14 (2017).

40. P. P. Rodrigo, Dispersal in primates: Variation in patterns and causes. *Metode Sci. Stud. J.* **5**, 35–41 (2015).

41. D. Hulce, X. Li, T. Snyder-Leiby, C. S. J. Liu, GeneMarker® genotyping software: Tools to increase the statistical power of DNA fragment analysis. *J. Biomol. Tech.* **22**, S35–S36 (2011).

42. S. D. E. Park, "Trypanotolerance in West African cattle and the population genetic effects of selection," thesis, University of Dublin, Dublin (2001).

43. P. A. Morin, K. E. Chambers, C. Boesch, L. Vigilant, Quantitative polymerase chain reaction analysis of DNA from noninvasive samples for accurate microsatellite genotyping of wild chimpanzees (*Pan troglodytes verus*). *Mol. Ecol.* **10**, 1835–1844 (2001).

44. A. Untergasser, I. Cutcutache, T. Koressaar, Y. Jian, B. C. Faircloth, M. Remm, S. G. Rozen, Primer3—New capabilities and interfaces. *Nucleic Acids Res.* **40**, e115 (2012).

45. H. Li, R. Durbin, Fast and accurate short read alignment with Burrows-Wheeler transform. *Bioinformatics* **25**, 1754–1760 (2009).

46. L. Hu, J. Long, Y. Lin, Z. Gu, H. Su, X. Dong, Z. Lin, Q. Xiao, N. Batbayar, B. Bold, L. Deutschová, S. Ganusevich, V. Sokolov, A. Sokolov, H. R. Patel, P. D. Waters, J. A. M. Graves, A. Dixon, S. Pan, X. Zhan, Arctic introgression and chromatin regulation facilitated rapid Qinghai-Tibet Plateau colonization by an avian predator. *Nat. Commun.* **13**, 6413 (2022).

47. H. Li, B. Handsaker, A. Wysoker, T. Fennell, J. Ruan, N. Homer, G. Marth, G. Abecasis, R. Durbin, 1000 Genome Project Data Processing Subgroup, The sequence alignment/map format and SAMtools. *Bioinformatics* **25**, 2078–2079 (2009).

48. A. McKenna, M. Hanna, E. Banks, A. Sivachenko, K. Cibulskis, A. Kernytsky, K. Garimella, D. Altshuler, S. Gabriel, M. Daly, M. A. DePristo, The Genome Analysis Toolkit: A MapReduce framework for analyzing next-generation DNA sequencing data. *Genome Res.* **20**, 1297–1303 (2010).

49. F. Pompanon, A. Bonin, E. Bellemain, P. Taberlet, Genotyping errors: Causes, consequences and solutions. *Nat. Rev. Genet.* **6**, 847–859 (2005).

50. L. F. K. Kuderna, H. Gao, M. C. Janiak, M. Kuhlwilm, J. D. Orkin, T. Bataillon, S. Manu, A. Valenzuela, J. Bergman, M. Rousselle, F. E. Silva, L. Agueda, J. Blanc, M. Gut, D. de Vries, I. Goodhead, R. A. Harris, M. Raveendran, A. Jensen, I. S. Chuma, J. E. Horvath, C. Hvilsom, D. Juan, P. Frandsen, J. G. Schraiber, F. R. de Melo, F. Bertuol, H. Byrne, I. Sampaio, I. Farias, J. Valsecchi, M. Messias, M. N. F. da Silva, M. Trivedi, R. Rossi, T. Hrbek, N. Andriaholinirina, C. J. Rabarivola, A. Zaramody, C. J. Jolly, J. Phillips-Conroy, G. Wilkerson, C. Abee, J. H. Simmons, E. Fernandez-Duque, S. Kanthaswamy, F. Shiferaw, D. Wu, L. Zhou, Y. Shao, G. Zhang, J. D. Keyyu, S. Knauf, M. D. Le, E. Lizano, S. Merker, A. Navarro, T. Nadler, C. C. Khor, J. Lee, P. Tan, W. K. Lim, A. C. Kitchener, D. Zinner, I. Gut, A. D. Melin, K. Guschanski, M. H. Schierup, R. M. D. Beck, G. Umapathy, C. Roos, J. P. Boubli, J. Rogers, K. K.-H. Farh, T. M. Bonet, A global catalog of whole-genome diversity from 233 primate species. *Science* **380**, 906–913 (2023).

51. G. Wang, M. Zhang, X. Wang, M. A. Yang, P. Cao, F. Liu, H. Lu, X. Feng, P. Skoglund, L. Wang, Q. Fu, Y. Zhang, Genomic approaches reveal an endemic subpopulation of gray wolves in Southern China. *iScience* **20**, 110–118 (2019).

52. T. J. Pemberton, C. Wang, J. Z. Li, N. A. Rosenberg, Inference of unexpected genetic relatedness among individuals in HapMap phase III. *Am. J. Hum. Genet.* **87**, 457–464 (2010).

53. S. Wahlund, Zusammensetzung von Populationen und Korrelationserscheinungen vom Standpunkt der Vererbungslehre aus betrachtet. *Hereditas* **11**, 65–106 (1928).

54. C. Do, R. S. Waples, D. Peel, G. M. Macbeth, B. J. Tillett, J. R. Ovenden, NeEstimator v2: Re-implementation of software for the estimation of contemporary effective population size (N_e) from genetic data. *Mol. Ecol. Resour.* **14**, 209–214 (2014).

55. G. Luikart, T. Antao, B. K. Hand, C. C. Muhlfeld, M. C. Boyer, T. Cosart, B. Trethewey, R. Al-Chockhachy, R. S. Waples, Detecting population declines via monitoring the effective number of breeders (N_b). *Mol. Ecol. Resour.* **21**, 379–393 (2021).

56. M. Kardos, G. Luikart, F. W. Allendorf, Measuring individual inbreeding in the age of genomics: Marker-based measures are better than pedigrees. *Heredity* **115**, 63–72 (2015).

57. S. Feng, Q. Fang, R. Barnett, C. Li, S. Han, M. Kuhlwilm, L. Zhou, H. Pan, Y. Deng, G. Chen, A. Gamauf, F. Woog, R. Prys-Jones, T. Marques-Bonet, M. T. P. Gilbert, G. Zhang, The genomic footprints of the fall and recovery of the crested ibis. *Curr. Biol.* **29**, 340–349 (2019).

58. M. Hasselgren, N. Dussex, J. von Seth, A. Angerbjörn, L. Dalén, K. Norén, Strongly deleterious mutations influence reproductive output and longevity in an endangered population. *Nat. Commun.* **15**, 8378 (2024).

59. H. Li, R. Durbin, Inference of human population history from individual whole-genome sequences. *Nature* **475**, 493–496 (2011).

60. S. Liu, M. M. Hansen, PSMC (pairwise sequentially Markovian coalescent) analysis of RAD (restriction site associated DNA) sequencing data. *Mol. Ecol. Resour.* **17**, 631–641 (2017).

61. X. Liu, Y.-X. Fu, Stairway Plot 2: Demographic history inference with folded SNP frequency spectra. *Genome Biol.* **21**, 280 (2020).

62. S. Boitard, W. Rodríguez, F. Jay, S. Mona, F. Austerlitz, Inferring population size history from large samples of genome-wide molecular data-an approximate Bayesian computation approach. *PLOS Genet.* **12**, e1005877 (2016).

63. E. Santiago, I. Novo, A. F. Pardiñas, M. Saura, J. Wang, A. Caballero, Recent demographic history inferred by high-resolution analysis of linkage disequilibrium. *Mol. Biol. Evol.* **37**, 3642–3653 (2020).

64. B. C. Haller, P. W. Messer, SLiM 3: Forward genetic simulations beyond the Wright–Fisher model. *Mol. Biol. Evol.* **36**, 632–637 (2019).

65. P. P. Khil, R. D. Camerini-Otero, Variation in patterns of human meiotic recombination. *Genome Dyn.* **5**, 117–127 (2009).

66. B. Y. Kim, C. D. Huber, K. E. Lohmueller, Inference of the distribution of selection coefficients for new nonsynonymous mutations using large samples. *Genetics* **206**, 345–361 (2017).

67. C. C. Kyriazis, R. K. Wayne, K. E. Lohmueller, Strongly deleterious mutations are a primary determinant of extinction risk due to inbreeding depression. *Evol. Lett.* **5**, 33–47 (2021).

68. J. Zhou, “The ecology and behavior traits of Hainan black-crested gibbon (*Nomascus hainanus*),” thesis, Northeast Normal University, Changchun (2008).

69. S. T. Turvey, K Traylor-Holzer, M. H. G Wong, J. V. Bryant, X. Zeng, X Hong, Y. Long, “International conservation planning workshop for the Hainan gibbon: Final report” (Zoological Society of London/IUCN SSC Conservation Breeding Specialist Group, 2015).

70. M. Nosrati, H. A. Nanaei, A. Javanmard, A. Esmailizadeh, The pattern of runs of homozygosity and genomic inbreeding in world-wide sheep populations. *Genomics* **113**, 1407–1415 (2021).

71. G. Bertorelle, F. Raffini, M. Bosse, C. Bortoluzzi, A. Iannucci, E. Trucchi, H. E. Morales, C. van Oosterhout, Genetic load: Genomic estimates and applications in non-model animals. *Nat. Rev. Genet.* **23**, 492–503 (2022).

72. T. Leroy, M. Rousselle, M. Tilak, A. E. Caizergues, C. Scornavacca, M. Recuerda, J. Fuchs, J. C. Illera, D. H. Swardt, G. Blanco, C. Thébaud, B. Milá, B. Nabholz, Island songbirds as windows into evolution in small populations. *Curr. Biol.* **31**, 1303–1310 (2021).

73. D. H. Alexander, J. Novembre, K. Lange, Fast model-based estimation of ancestry in unrelated individuals. *Genome Res.* **19**, 1655–1664 (2009).

74. S. Purcell, B. Neale, K. Todd-Brown, L. Thomas, M. A. R. Ferreira, D. Bender, J. Maller, P. Sklar, P. I. W. de Bakker, M. J. Daly, P. C. Sham, PLINK: A tool set for whole-genome association and population-based linkage analyses. *Am. J. Hum. Genet.* **81**, 559–575 (2007).

75. W. He, L. Xu, J. X. Wang, Y. Zhen, J. Yi, S. Tai, J. Yang, X. Fang, VCF2PCACluster: A simple, fast and memory-efficient tool for principal component analysis of tens of millions of SNPs. *BMC bioinformatics* **25**, 173 (2024).

76. L. Xu, W. He, S. Tai, X. Huang, M. Qin, X. Liao, Y. Jing, J. Yang, X. Fang, J. Shi, N. Jin, VCF2Dis: An ultra-fast and efficient tool to calculate pairwise genetic distance and construct population phylogeny from VCF files. *GigaScience* **14**, giaf032 (2025).

77. Y. Guo, D. Peng, L. Han, T. Liu, G. Li, P. A. Garber, J. Zhou, Mitochondrial DNA control region sequencing of the critically endangered Hainan gibbon (*Nomascus hainanus*) reveals two female origins and extremely low genetic diversity. *Mitochondrial. DNA B Resour.* **6**, 1355–1359 (2021).

78. L. Excoffier, N. Marchi, D. A. Marques, R. Matthey-Doret, A. Gouy, V. C. Sousa, fastsimcoal2: Demographic inference under complex evolutionary scenarios. *Bioinformatics* **37**, 4882–4885 (2021).

79. R. N. Gutenkunst, R. D. Hernandez, S. H. Williamson, C. D. Bustamante, Inferring the joint demographic history of multiple populations from multidimensional SNP frequency data. *PLOS Genet.* **5**, e1000695 (2009).

80. R Core Team, "R: A language and environment for statistical computing" (R Foundation for Statistical Computing, 2020); www.R-project.org/.

81. M. Kardos, R. S. Waples, Low-coverage sequencing and Wahlund effect severely bias estimates of inbreeding, heterozygosity and effective population size in North American wolves. *Mol. Ecol.* **34**, e17415 (2024).

82. M. Boehnke, N. J. Cox, Accurate inference of relationships in sib-pair linkage studies. *Am. J. Hum. Genet.* **61**, 423–429 (1997).

83. M. P. Epstein, W. L. Duren, M. Boehnke, Improved inference of relationship for pairs of individuals. *Am. J. Hum. Genet.* **67**, 1219–1231 (2000).

84. H. Deng, M. Zhang, J. Zhou, Recovery of the critically endangered Hainan gibbon *Nomascus hainanus*. *Oryx* **51**, 161–165 (2017).

85. Z. Liu, Y. Zhang, H. Jiang, C. Southwick, Population structure of *Hylobates concolor* in Bawanglin Nature Reserve, Hainan, China. *Am. J. Primatol.* **19**, 247–254 (1989).

86. Z. Liu, S. Yu, X. Yuan, Resources of the Hainan black gibbon and its present situation. *Chin. Wildl.* **6**, 1–4 (1984).

87. Z. Liu, Z. H. Jiang, Y. Zhang, Y. Liu, C. Tigon, D. Manry, C. Southwick, Field report on the Hainan gibbon. *Primate Conserv.* **8**, 49–50 (1987).

88. W. Wei, X. Wang, F. Claro, Y. Ding, A. Souris, C. Wang, C. Wang, R. Berzins, The current status of the Hainan black-crested gibbon *Nomascus* sp. cf. *nasutus hainanus* in Bawangling National Nature Reserve, Hainan, China. *Oryx* **38**, 452–456 (2004).

89. X. Song, H. Jiang, J. Zhang, Q. Chen, C. Wang, W. Lin, Chniese, “A survey of the Hainan gibbon (Hylobates concolor hainanus) in Hainan Island,” in Zoological Studies in China, (China Academic Journal Electronic Publishing House, 1999), pp. 696–701.

90. S. T. Turvey, J. V. Bryant, C. Duncan, M. H. G. Wong, Z. Guan, H. Fei, C. Ma, X. Hong, H. C. Nash, B. P. L. Chan, Y. Xu, P. Fan, How many remnant gibbon populations are left on Hainan? Testing the use of local ecological knowledge to detect cryptic threatened primates. *Am. J. Primatol* **79**, e22593 (2017).

91. B. P. L. Chan, Y. F. P. Lo, Y. Mo, New hope for the Hainan gibbon: Formation of a new group outside its known range. *Oryx* **54**, 296–296 (2020).

92. A. Määttä, T. DiColandrea, K. Groot, F. M. Watt, Gene targeting of *envoplakin*, a cytoskeletal linker protein and precursor of the epidermal cornified envelope. *Mol. Cell. Biol.* **21**, 7047–7053 (2001).

93. L. M. Sevilla, R. Nachat, K. R. Groot, J. F. Klement, J. Uitto, P. Djian, A. Määttä, F. M. Watt, Mice deficient in *involucrin*, *envoplakin*, and *periplakin* have a defective epidermal barrier. *J. Cell Biol.* **179**, 1599–1612 (2007).

94. M. Grill, T. E. Syme, A. L. Nocon, A. Z. X. Lu, D. Hancock, S. Rose-John, L. L. Campbell, Strawberry notch homolog 2 is a novel inflammatory response factor predominantly but not exclusively expressed by astrocytes in the central nervous system. *Glia* **63**, 1738–1752 (2015).

95. H. Zhang, Q. Zhang, J. Tu, Q. You, L. Wang, Dual function of protein phosphatase 5 (PPP5C): An emerging therapeutic target for drug discovery. *Eur. J. Med. Chem.* **254**, 115350 (2023).

96. M. A. Lalli, J. Jang, J. C. Park, Y. Wang, E. Guzman, H. Zhou, M. Audouard, D. Bridges, K. R. Tovar, S. M. Papuc, A. C. Tutulan-Cunita, Y. Huang, M. Budisteanu, A. Arghir, K. S. Kosik, Haploinsufficiency of *BAZ1B* contributes to Williams syndrome through transcriptional dysregulation of neurodevelopmental pathways. *Hum. Mol. Genet.* **25**, 1294–1306 (2016).

97. N. K. Noren, E. B. Pasquale, Paradoxes of the EphB4 receptor in cancer. *Cancer Res.* **67**, 3994–3997 (2007).

98. J. A. Sake, M. A. Selo, L. Burtnyak, H. E. Dähnhardt, C. Helbet, S. Mairinger, O. Langer, V. P. Kelly, C. Ehrhardt, Knockout of *ABCC1* in NCI-H441 cells reveals CF to be a suboptimal substrate to study MRP1 activity in organotypic in vitro models. *Eur. J. Pharm. Sci.* **181**, 106364 (2023).

99. M. K. Montgomery, J. Bayliss, S. Keenan, S. Rhost, S. B. Ting, M. J. Watt, The role of Ap2a2 in PPARα-mediated regulation of lipolysis in adipose tissue. *FASEB J.* **33**, 13267–13279 (2019).

100. T. V. Morozova, T. F. C. Mackay, R. H. A. Anholt, Genetics and genomics of alcohol sensitivity. *Mol. Genet. Genomics* **289**, 253–269 (2014).

101. J. Liu, X. Luo, Y. Xu, J. Gu, F. Tang, Y. Jin, H. Li, Single-stranded DNA binding protein Ssbp3 induces differentiation of mouse embryonic stem cells into trophoblast-like cells. *Stem Cell Res. Ther.* **7**, 79 (2016).

102. Y. Zhang, W. Lui, CXADR: From an essential structural component to a vital signaling mediator in spermatogenesis. *Int. J. Mol. Sci.* **24**, 1288 (2023).

103. J. Chemnitz, D. Pieper, L. Stich, U. Schumacher, S. Balabanov, M. Spohn, A. Grundhoff, A. Steinkasserer, J. Hauber, E. Zinser, The acidic protein rich in leucines Anp32b is an immunomodulator of inflammation in mice. *Sci. Rep.* **9**, 4853 (2019).

104. S. Khadka, L. Vien, P. Leonard, L. Bover, F. Muller, Generation and validation of an anti-human PANK3 mouse monoclonal antibody. *Biomolecules* **12**, 1323 (2022).

105. M. M. Klairmont, W. L. Carroll, L. Aifantis, C. Y. Park, High *ORM1* expression marks a subset of genetically Adverse-Risk B-ALL characterized by MDSC enrichment, T-Cell dysfunction, and inferior overall survival. *Blood* **140**, 6360–6360 (2022).

106. A. M. Alam, Nipah virus, an emerging zoonotic disease causing fatal encephalitis. *Clin. Med.* **22**, 348 (2022).

107. X. Liu, J. Peng, Y. Zhou, B. Xie, J. Wang, Silencing RRM2 inhibits multiple myeloma by targeting the Wnt/β catenin signaling pathway. *Mol. Med. Rep.* **20**, 2159–2166 (2019).
